# Isolated *C. elegans* germ nuclei exhibit distinct genomic profiles of histone modification and gene expression

**DOI:** 10.1186/s12864-019-5893-9

**Published:** 2019-06-17

**Authors:** Mei Han, Guifeng Wei, Catherine E. McManus, LaDeana W. Hillier, Valerie Reinke

**Affiliations:** 1 Department of Genetics, Yale University, New Haven, CT 06520 USA; 2 Developmental Epigenetics, Department of Biochemistry, University of Oxford, Oxford, OX1 3QU UK; 3 Department of Genome Sciences, University of Washington School of Medicine, Seattle, Washington, 98195 USA

**Keywords:** *C. elegans*, Germ cells, Gene expression, Genomics, Method development

## Abstract

**Background:**

The wide variety of specialized permissive and repressive mechanisms by which germ cells regulate developmental gene expression are not well understood genome-wide. Isolation of germ cells with high integrity and purity from living animals is necessary to address these open questions, but no straightforward methods are currently available.

**Results:**

Here we present an experimental paradigm that permits the isolation of nuclei from *C. elegans* germ cells at quantities sufficient for genomic analyses. We demonstrate that these nuclei represent a very pure population and are suitable for both transcriptome analysis (RNA-seq) and chromatin immunoprecipitation (ChIP-seq) of histone modifications. From these data, we find unexpected germline- and soma-specific patterns of gene regulation.

**Conclusions:**

This new capacity removes a major barrier in the field to dissect gene expression mechanisms in the germ line of *C. elegans*. Consequent discoveries using this technology will be relevant to conserved regulatory mechanisms across species.

**Electronic supplementary material:**

The online version of this article (10.1186/s12864-019-5893-9) contains supplementary material, which is available to authorized users.

## Background

Establishing tissue-specific gene expression programs during development requires dynamic, highly coordinated gene regulation, often over extended genomic regions. The *C. elegans* germ line is an ideal microcosm to explore complex gene expression regulatory mechanisms. These germ cells deploy diverse, tightly controlled gene regulatory programs to drive proliferation, meiosis and gamete differentiation, yet retain the ability to reactivate totipotency in the zygote [1]. They must therefore repress somatic gene expression, which could lead to inappropriate or premature differentiation [2]. Indeed, ectopic activation of somatic programs readily transforms germ cells to neurons, intestine, and muscle [3, 4]. Germ cells exhibit long-range regulation as well, across multi-megabase-long piRNA gene clusters [5] and over the entire X chromosome [6]. All of these complex events must be precisely coordinated to permit the production of hundreds of viable embryos in each hermaphrodite in just a few short days of reproductive capacity.

Chromatin-based, post-transcriptional, and small RNA mechanisms play a central role in modulating transcript and protein abundance in the germ line [7]. For example, the conserved Rb/E2F regulatory complex is critical for establishing distinct germline and somatic gene expression programs [8–11]. Additionally, germ cells are transformed to somatic cells in vivo either by disrupting chromatin regulation via forced expression of a somatic transcription factor concomitant with loss of chromatin factor LIN-53 [4], or by disrupting post-transcriptional regulation through loss of mRNA-binding proteins MEX-3 and GLD-1 [12] or loss of germ granules [13, 14]. Distinct small RNA pathways selectively target transcripts either for degradation or protection in the cytoplasm, and ultimately alter chromatin state as well. Disrupting the feedback from cytoplasm to nucleus causes germ cells to gradually lose their identity over multiple generations [15].

To fully investigate these regulatory mechanisms, genome-scale assays are necessary. However, in many species, it is difficult to isolate sufficient germ cells at key developmental times due to their relative scarcity and sequestration within various somatic niches. In *C. elegans*, germ cells are prominent in number and location relative to somatic cells, but to date, no methods have been developed to purify them from living animals, in part because these cells share cytoplasm via cellular bridges and therefore exist in a syncytium. Moreover, thousands of animals are required to provide sufficient material for most assays, eliminating the option of gonad dissection. Although proteins can be epitope-tagged and expressed specifically in the germ line for tissue-specific ChIP-seq [9], many other applications such as histone modification profiling and chromatin organization assays require pure chromatin preparations. Histone modifications and chromatin states have therefore been measured primarily in whole animal or embryo preparations, which mix somatic and germ cell populations and complicate interpretation of the data. Here we report a simple method that circumvents this limitation and produces populations of germ nuclei at ~ 90% purity, with yields sufficient for biochemical and genomic analyses. The method does not require specialized transgenic strains or growth conditions, and can be readily utilized with minimal troubleshooting. We show that these isolated germ nuclei (IGN) can be used for both RNA-seq and histone modification ChIP-seq, and exhibit expected patterns of germline gene regulation. In addition, these analyses point to some distinct mechanisms of gene regulation in germ cells compared to somatic cells. In sum, the isolation procedure presented here is straightforward and easily adaptable to any worm strain with appreciable numbers of germ cells, and we fully expect that these nuclei will be useful for additional genomic assays, including chromatin capture conformation, nucleosome accessibility, and small RNA-seq, among others.

## Results

### A highly efficient method to isolate germ nuclei from *C. elegans* adults

**Fig. 1:**
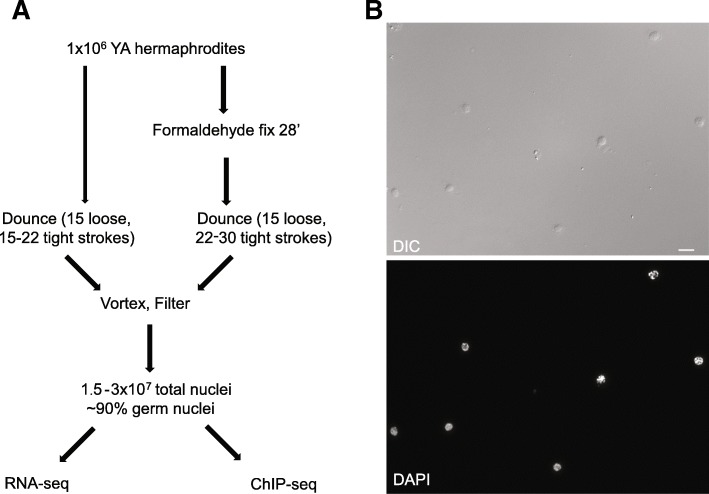
A simple method to isolate germ nuclei from *C. elegans.* **a** Schematic representation of the method to isolate germ nuclei from *C. elegans.* Approximately 1 × 10^6^ young adult hermaphrodites were collected for nuclei isolation for each experiment. For RNA-seq, worms were homogenized with 15 loose and 15–22 tight Dounce strokes after collection. For ChIP-seq, worms were fixed with 2% formaldehyde for 28 min before homogenization with 15 loose and 22–30 tight Dounce strokes (see Materials and Methods). A typical yield is 1.5–3 × 10^7^ nuclei from 1 × 10^6^ young adult hermaphrodites. **b** Representative image of isolated total nuclei from young adult worms stained with DAPI. Scale bar, 10 μm

We have developed a novel, simple procedure to isolate germ nuclei at a scale that permits biochemical analyses (Fig. 1a). Based on a previous report in which intestine nuclei could be isolated from whole animals [16], we determined conditions in which germ nuclei are preferentially released from the syncytial gonad by gentle Dounce homogenization and vortexing of whole adult animals, followed by filtration to remove cellular (somatic) debris (see Materials and Methods). The method takes approximately three hours from worm harvest to nuclear pellet and results in 1.5-3 × 10^7^ nuclei from ~ 1 × 10^6^ worms. Isolated nuclei show relatively uniform size and intact nuclear structure based on DAPI staining (Fig. 1b).

**Fig. 2:**
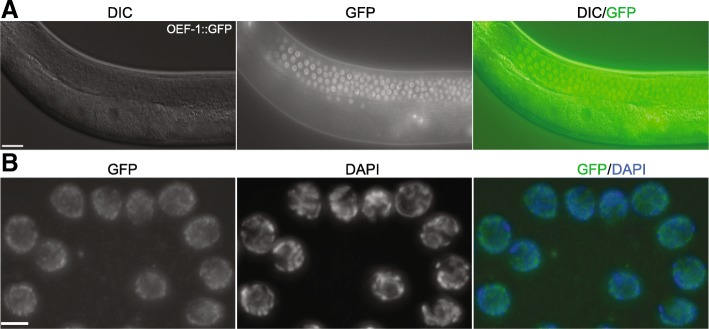
Quantification of isolated germline nuclei. **a** Young adult OEF-1::GFP transgenic worms with germ line expression. OEF-1 is detected specifically on autosomes in mitotic and pachytene nuclei and disappears at the onset of oogenesis [17]. **b** Isolated nuclei from OEF-1::GFP young adult worms were immunostained with GFP (green) and stained with DAPI (blue). Nuclei stained with both GFP and DAPI were designated germline nuclei (91.02% of total nuclei, *n* = 2127). Two independent biological replicates were performed. Scale bars, 20 μm in (A), 5 μm in (**b**)

To calculate the percentage of isolated nuclei that are from germ cells, we performed the isolation procedure on a transgenic strain that expresses OEF-1::GFP. OEF-1 is a novel germline factor present specifically in mitotic and pachytene nuclei that disappears abruptly at the onset of oogenesis [17] (Fig. 2a). Immunostaining of isolated nuclei from this strain with anti-GFP indicates that 91% are positive for OEF-1 (Fig. 2b and Additional file 9: Table S1). We also used a second germline-specific transgenic strain, AZ212, that expresses GFP::H2B specifically in the germ line under the control of the *pie-1* promoter [18] (Additional file 1: Figure S1). In this strain, GFP::H2B is expressed in the nuclei of germ cells at all developmental stages, including oocytes. However, we found a staining frequency (89%) in isolated nuclei similar to that of OEF-1::GFP, which suggests that most of the nuclei obtained are from the distal and/or medial gonad. Indeed, many of the isolated nuclei appear to be in the pachytene stage based on chromosome morphology visible by DAPI staining (Fig. 2b and Additional file 1: Figure S1B). Many of the unstained nuclei exhibit extremely condensed DNA, and we suspect that they represent sperm that are released during the disruption protocol and pass through the size filters (Additional file 2: Figure S2). Thus, the actual percentage of somatic nuclei present in the population is likely much less than 10%.

We have performed this isolation protocol on both unfixed and fixed animals, depending on the downstream application. Fixation slightly increases the number of Dounce strokes necessary to break open the animals but does not otherwise impair the procedure (Fig. 1; Materials and Methods). One of the main advantages of this approach is that it does not require any specialized transgenic system or subsequent affinity purification, and it is applicable to any transgenic or mutant strain with reasonable numbers of germ nuclei. Thus, this method is rapid, simple, reproducible, and adaptable. We use the abbreviation “IGN” to refer to these isolated germ nuclei. As described below, we have performed IGN-RNA-seq, as well as IGN-ChIP-seq for two histone modifications, demonstrating that the nuclei are amenable to a wide variety of genomic assays.

### IGN expression analysis

**Fig. 3:**
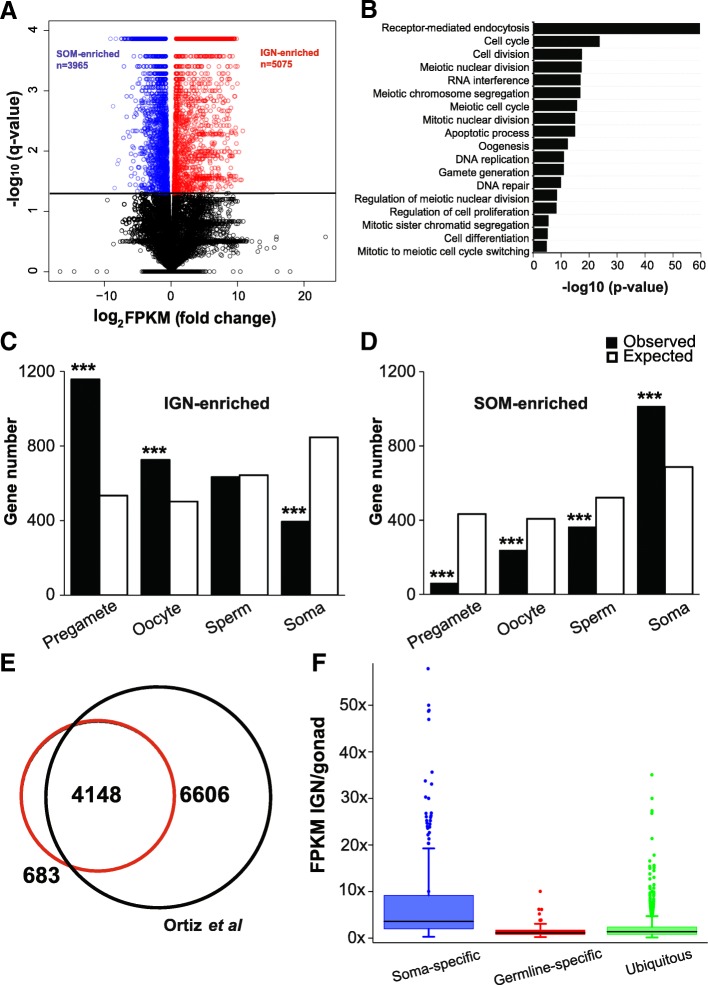
Expression profiling of *C. elegans* isolated germline nuclei. RNA from two independent replicates of wild type IGN and from SOM (*glp-1(q224)* young adults) was analyzed for expression profiling. **a** Volcano plot showing –log10 of q-value against log2 of fold change for each gene. The number of genes that were significantly up-regulated in SOM or IGN are indicated. Black line marks the significance cutoff of q = 0.05 (Y axis). Blue circles indicate SOM-enriched transcripts and red circles indicate IGN-enriched transcripts. **b** The most significant Gene Ontology Biological Process terms of the 5075 IGN-enriched transcripts. **c-d** Bar graphs indicating expected and observed number of genes (Y axis) in different gene categories [22] (X axis) for IGN-enriched transcripts (**c**) and SOM-enriched transcripts (**d**). Asterisks indicate significantly more genes than expected (hypergeometric test, *p*-value< 1 × 10^− 5^ [**], p-value< 1 × 10^− 10^ [***]). **e** Overlap of 4831 IGN-enriched coding transcripts with previously-identified germline-expressed transcripts in dissected gonads [23]. **f** The ratio of IGN FPKM to dissected gonad FPKM for genes previously defined as having soma-specific, germline-specific, and ubiquitous expression [24]

We first performed total RNA-seq with ribosomal RNA depletion on IGN from wild type (N2) young adults. To permit direct comparison to somatic gene expression, we also performed whole-animal RNA-seq on *glp-1(q224)* young adults, which have a temperature-sensitive mutation in the GLP-1/Notch receptor. At the restrictive temperature, *glp-1(q224)* mutants lack all germ cells except for a few mature sperm, and thus represent only somatic tissues (which we abbreviate as SOM throughout the manuscript) [19]. Two independent RNA-seq experiments were analyzed for each genotype using HISAT2 [20] and Cuffdiff [21]. On average, 24 million paired-end sequenced reads were mapped to the *C. elegans* genome (ce10) per sample. With Cuffdiff, transcript abundance is calculated as Fragments Per Kilobase of transcript per Million mapped reads (FPKM). Separately, we also performed read count-based analysis (DESeq2), but found little difference between the two methods (Additional file 3: Figure S3) and so present our analyses as FPKM. We observed many obvious differences between IGN and SOM profiles in genome browser views (Additional file 4: Figure S4A and S4B). We therefore identified differentially expressed genes between IGN and SOM with a q value less than 0.05, which equals 1.36-fold or greater difference in expression. These analyses identified 5075 genes with IGN-enriched expression and 3965 genes with SOM-enriched expression (Fig. 3a and Additional file 4: Figure S4C; Additional file 10). Within these groups, 244 and 46 noncoding lncRNAs have higher expression in IGN and SOM, respectively. We examined the Gene Ontology (GO) biological process terms for IGN-enriched transcripts. Strikingly, most terms are related to the mitotic or meiotic cell cycle and gamete generation, indicating that genes with higher expression in IGN relative to SOM are significantly associated with germline-related functions (Fig. 3b). In particular, the most significant category, receptor-mediated endocytosis, reflects the requirement for this class of genes in the uptake of yolk from the intestine by oocytes [25].

To determine how well the IGN-enriched dataset reflects known germline-expressed or germline-enriched gene expression profiles, we made several comparisons with published datasets. First, we compared our dataset to a published expression profile from dissected hermaphrodite gonads that identified 10,754 genes as expressed in that tissue [23]. Of the 5075 genes with IGN-enriched expression, 4831 are coding and 244 are non-coding genes. We found that 4148 (~ 86%) out of the 4831 are present in the dissected gonad dataset (Fig. 3e; Additional file 11). Of the 683 genes present exclusively in the IGN dataset, many are encoded by genes with known germline function, such as *hal-2*, *rec-1*, *mes-6*, *snpc-4*, *vbh-1*, etc. Many others have quite low expression and might be a consequence of different experimental design (e.g. poly-A purification vs ribo-depletion strategies) or different cutoffs in the analysis. Conversely, a large number (6606) of genes are present exclusively in the dissected gonad dataset. This observation is not surprising because the dissected gonad dataset represents a comprehensive set of germline-expressed genes regardless of somatic gene expression, whereas the IGN-enriched dataset excludes genes expressed at similar or higher levels in the soma. Consistent with this possibility, GO analysis indicates that the 6606 genes represented exclusively in the dissected gonad dataset mainly contribute to fundamental cellular processes, including oxidation-reduction, lipid metabolism, transport, proteolysis etc., which occur in most or all cell types and are not expected to be especially enriched in germ cells (Additional file 5: Figure S5).

Second, we compared genes with IGN- and SOM-enriched expression to genes previously placed in distinct expression categories: pre-gametic germ cells, oocytes, sperm, and the soma [22]. Genes in the IGN-enriched dataset are over-represented among the pre-gamete and oocyte but not the sperm category, and under-represented in the soma category (Fig. 3c; Additional file 11). Conversely, genes in the SOM-enriched dataset are over-represented in the soma category and under-represented in the germline-related categories (Fig. 3d and Additional file 11).

The IGN RNA-seq profile primarily represents the RNA population from isolated germ nuclei, whereas previously published germline profiling experiments, such as those from dissected whole gonads, include both nuclear and cytoplasmic populations. Relative to the cytoplasm, nuclear RNA pools should be enriched for primary or partially processed transcripts. Overall, more sequencing reads mapped to introns in IGN (16.8%) relative to SOM (10.2%), and introns are more abundantly represented per gene in IGN as well (Additional file 6: Figure S6). IGN therefore have an increased level of partially-processed transcripts as expected (see Additional file 4: Figure S4B as an example).

Because IGN have increased representation of primary or partially processed transcripts, we were interested in whether we could detect evidence of somatic gene expression in IGN. Previous experiments demonstrate that wild type germ cells inhibit somatic gene expression post-transcriptionally, implying that germ cells must initially transcribe these genes [12–14]. If such somatic transcripts are indeed expressed in germ nuclei and subsequently degraded in the cytoplasm, we hypothesized that somatic transcripts would have higher expression in IGN (nuclear RNA pool only) relative to the whole gonad (nuclear plus cytoplasmic RNA pool), whereas germline-expressed genes would not. To test this idea, we compared the ratio of IGN FPKM to whole gonad FPKM for genes previously defined as having soma-specific, germline-specific, and ubiquitous expression [24]. In line with our prediction, soma-specific transcripts are present at higher abundance (mean: 9.8x IGN/gonad) in wild type germ nuclei relative to whole gonads, whereas germline-specific and ubiquitous transcripts have much lower ratios (1.3x and 1.8x, respectively) (Fig. 3f). This result suggests that indeed a set of soma-specific transcripts are initially expressed in germ nuclei but quickly degraded post-transcriptionally. Thus, even though IGN contain RNA populations that broadly mirror expected germline-specific gene expression profiles, these results indicate that IGN samples are potentially suitable to examine RNAs largely restricted to the nucleus as well. As such, IGN are likely to provide new insights into germline regulatory mechanisms.

### Chromatin modification profiling of germ nuclei

We next tested whether IGN could be used to profile histone modifications specifically in germ nuclei. We purified chromatin from IGN, as well as from *glp-1* mutant young adults to represent somatic tissues (SOM). We initially used ChIP-qPCR to test a handful of loci for the presence of the H3K27ac modification, which marks active regulatory elements. Two loci correspond to the upstream regulatory regions of genes with known germline-specific expression, *him-3* [26] and *oef-1* [17]. Three loci were selected based on existing H3K27ac ChIP-chip data from the ENCODE project at the L3 stage of development (GSM624432 and GSM624433) [27] to serve as positive (*C37H5.15*) and negative (*elt-2, myo-3*) controls. Both *him-3* and *oef-1* upstream regions showed significant enrichment (10–30x) for H3K27ac in IGN (Additional file 7: Figure S7).

We therefore selected two histone modifications that are highly associated with active enhancers/promoters and gene expression, H3K27ac and H3K4me3 [28–30]. We performed ChIP-seq on IGN and SOM chromatin collected in biological duplicate for each modification, and identified significant peaks using MACS2 (see Materials and Methods). For simplicity, we refer to these datasets as IGN-H3K27ac, IGN-H3K4me3, SOM-H3K27ac, and SOM-H3K4me3.

**Fig. 4:**
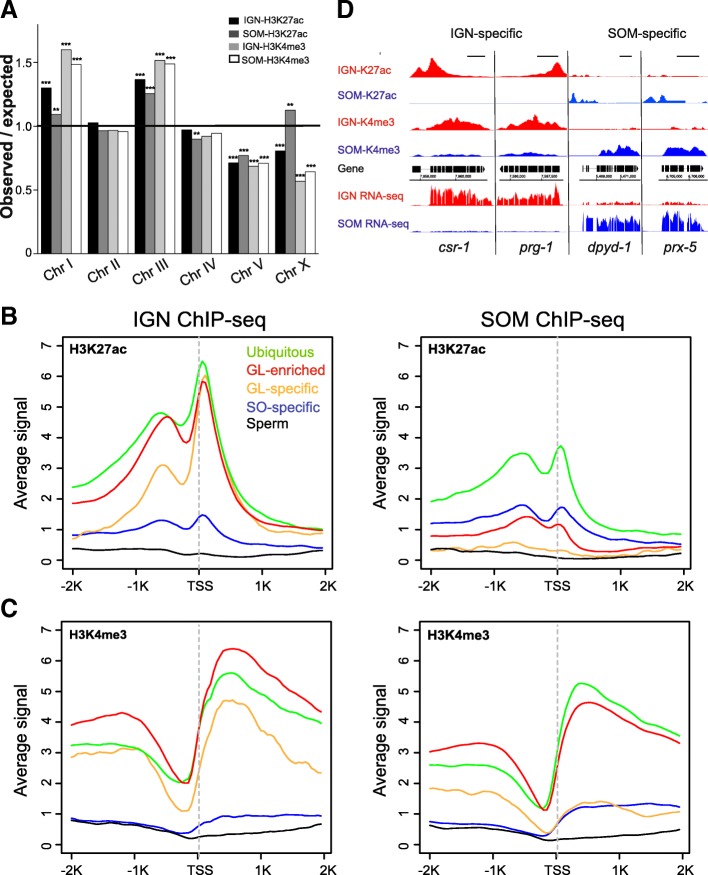
H3K27ac and H3K4me3 exhibit tissue-specific profiles in IGN and SOM. **a** Distribution of H3K27ac and H3K4me3 peaks across chromosomes relative to chromosome length for IGN- or SOM-H3K27ac ChIP-seq and IGN- or SOM-H3K4me3 ChIP-seq datasets. Statistically significant deviations from the expected value of 1 (indicated by black line) are indicated by asterisks (Pearson’s chi-square test, p-value< 1 × 10^− 3^ [**], p-value< 1 × 10^− 10^ [***]). (**b** and **c**) Metagene analysis of the distribution of average IGN- and SOM-H3K27ac ChIP-seq signal (**b**) and IGN- and SOM-H3K4me3 ChIP-seq signal (**c**) across bodies of genes in indicated categories [13]. **d** Example ChIP-seq and RNA-seq tracks across the IGN-specific genes *csr-1* and *prg-1* and SOM-specific genes *dpyd-1* and *prx-5*. IGN signals are indicated in red and SOM signals are indicated in blue. Scale bars, 1 kb

We first determined whether peaks in each dataset were consistent with previously described patterns. Immunostaining of dissected gonads has indicated that activating histone modifications are primarily associated with autosomes and depleted from the X chromosome in germ cells, where the X is largely silenced [24, 31]. As expected, fewer IGN-H3K27ac and IGN-H3K4me3 peaks than expected are observed on the X (Fig. 4a). By contrast, the X had more peaks than expected in the SOM-H3K27ac dataset. The SOM-H3K4me3 dataset also exhibited an increase in X-linked peaks relative to IGN-H3K4me3, although overall this number was lower than expected (see Discussion). Certain autosomes had significant variations from expected values of peaks, but for a given autosome, the trend was the same for both IGN and SOM. For example, Chr V had fewer than expected peaks in all four datasets, likely due to the preponderance of unexpressed chemoreceptor pseudogenes on that chromosome [32].

We next used metagene analysis to examine chromatin profiles for genes assigned to one of five previously published expression categories: ubiquitous, germline-specific, germline-enriched, soma-specific, and sperm [13] (Fig. 4b and c). Genes classified as ubiquitous showed abundant H3K27ac and H3K4me3 enrichment in IGN and SOM, indicating a broadly active chromatin state in most cells. Conversely, the lack of either modification at genes in the sperm category in both IGN and SOM is consistent with the fact that such genes, which are normally expressed only in the L4 germline [33], are not actively transcribed in the adult IGN or SOM. The most striking changes between cell types were observed for the germline-enriched and germline-specific gene categories. Genes in the germline-specific category displayed much higher levels of both H3K27ac and H3K4me3 in IGN than in SOM. Interestingly, while the germline-enriched category also had substantially higher H3K27ac in IGN relative to SOM, the H3K4me3 levels were only mildly higher. Another unexpected observation is that genes in the soma-specific category exhibited only minimal increases in overall H3K27ac and H3K4me3 levels in SOM relative to IGN. This finding is perhaps related to the observation that somatic genes are minimally transcribed in the germline (see Fig. 3f) and thus might be expected to display some H3K4me3 in IGN, although it is still notable that the levels of SOM-H3K4me3 are surprisingly low. Altogether, these results indicate that the chromatin modification profiles are generally consistent with expectations based on previously published data, but that modifications do not appear to behave in the same manner in IGN and SOM.

We therefore explored more closely tissue-specific binding events for both histone modifications. Across all chromosomes, tissue-specific H3K27ac and H3K4me3 ChIP-seq signals were easily distinguished between IGN and SOM in the genome browser. Many genes known to have germline-specific expression, such as *csr-1* and *prg-1*, exhibit high levels of IGN-H3K27ac and IGN-H3K4me3 at or near their transcription start sites and minimal to no signal in SOM, while many genes such as *dpyd-1* and *prx-5* exhibit the opposite pattern (Fig. 4d). As previously demonstrated [34], H3K4me3 was present in gene bodies as well as at promoters (Additional file 8: Figure S8). We assigned significant H3K27ac and H3K4me3 peaks to neighboring candidate genes genome-wide (Additional files 12, 13, 14, and 15; Materials and Methods). IGN-H3K27ac was present at significant levels at 3585 genes and SOM-H3K27ac at 3153 genes, while IGN-H3K4me3 was present at 6853 genes and SOM-H3K4me3 at 7727 genes.

**Fig. 5:**
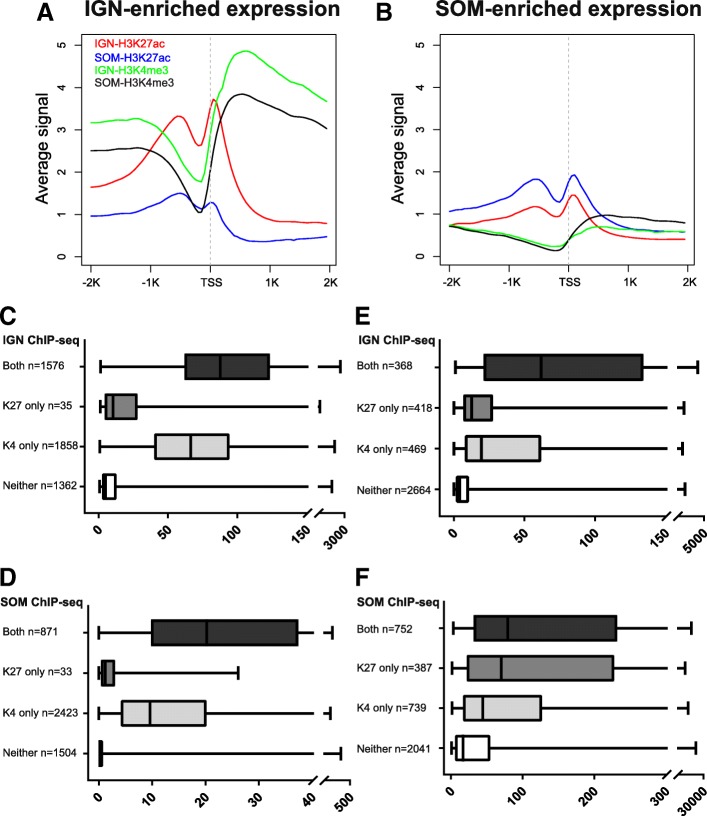
Genes with tissue-enriched expression exhibit distinct H3K27ac and H3K4me3 modifications in IGN and SOM. **a** and **b** Metagene analysis shows the average signal distribution of H3K27ac ChIP-seq and H3K4me3 ChIP-seq across gene bodies for 5075 IGN-enriched transcripts (**a**) and 3965 SOM-enriched transcripts (**b**). **c-f** The contribution of H3K27ac and H3K4me3 modifications (Y axis) to transcript abundance (FPKM, X axis) for genes with tissue-enriched expression in IGN and SOM (Additional files 12 and 13, Additional files 16 and 17). **c** IGN-H3K27ac and IGN-H3K4me3 modification status for genes with IGN-enriched expression. **d** SOM-H3K27ac and SOM-H3K4me3 modification status for genes with IGN-enriched expression. **e** IGN-H3K27ac and IGN-H3K4me3 modification status for genes with SOM-enriched expression. **f** SOM-H3K27ac and SOM-H3K4me3 modification status for genes with SOM-enriched expression

Because both modifications are associated with active genes, we analyzed the relationship between the presence of the two histone modifications and gene expression for IGN and SOM. We first performed metagene analysis of histone modification profiles for genes with IGN-enriched and SOM-enriched expression (Fig. 5a and b). As expected, genes with IGN-enriched expression have higher H3K27ac and H3K4me3 levels in IGN versus SOM, while genes with SOM-enriched expression have the opposite pattern. Similar to what was observed for published gene expression data (Fig. 4b and c), genes with IGN-enriched expression have higher levels of SOM-H3K4me3, compared to genes with SOM-enriched expression (compare black lines in 5A and 5B), suggesting that the presence of H3K4me3 is relatively uncoupled from gene expression in the soma. Possible explanations for this observation are provided in the Discussion.

To further determine how well transcript abundance correlated with the presence of histone modifications, we plotted FPKM for genes with IGN- or SOM-enriched expression, categorized by the presence of one or both modifications in either IGN or SOM (Fig. 5c-f). For genes with IGN-enriched expression, IGN-H3K4me3 was more associated with transcript abundance than H3K27ac (Fig. 5c). Moreover, only 35 genes in this category exhibited H3K27ac in the absence of H3K4me3, while 1858 were marked by H3K4me3 without H3K27ac. Examination of SOM-H3K4me3 and SOM-H3K27ac for genes with IGN-enriched expression showed similar trends (Fig. 5d), although overall expression levels were lower (compare X axes in 5C and 5D). Interestingly, many genes marked with both modifications in IGN appear to “lose” H3K27ac in SOM (1576 vs 871) while retaining H3K4me3 only (1858 vs 2423). Thus, even though H3K4me3 correlates better with transcript abundance, H3K27ac alters more substantially between the two tissues. Notably, even in SOM, the majority of genes with IGN-enriched expression retain at least one modification (3327/4831, or 69%), possibly because this dataset includes broadly expressed genes with only mild enrichment in germ cells.

By contrast, these trends are not so apparent for genes with SOM-enriched expression (Fig. 5e and f). In particular, transcript abundance of genes with SOM-enriched expression better correlates with SOM-H3K27ac than with SOM-H3K4me3 (Fig. 5f), and it is notable that more than half of these genes (2041/3919, or 52%) lack either modification in SOM. Indeed, these histone modifications are clearly not the only contributors to expression, as “baseline” expression is higher in the corresponding tissue for each dataset even in the absence of either mark (compare the “Neither” category between 5C and 5D for genes with IGN-enriched expression and between 5E and 5F for genes with SOM-enriched expression). Altogether, these observations indicate that the relationship between activating histone modifications and gene expression is much more straightforward in the germ line compared to the soma.

In sum, this analysis indicates that IGN can be used to isolate chromatin and perform ChIP-seq to identify histone modification profiles that are present specifically in adult germ nuclei, and clarify tissue-specific relationships between chromatin status and expression.

## Discussion

Here we present a novel method for isolating germ nuclei (IGN) from *C. elegans* adult hermaphrodites in quantities sufficient for genomic analyses. We demonstrate that IGN preparations contain at least ~ 90% germ nuclei under conditions in which animals are fixed prior to Dounce treatment. Moreover, we establish that IGN are suitable for genomic assays such as RNA-seq and histone modification ChIP-seq, and display profiles consistent with prior analyses of germline gene expression. Importantly, this procedure does not require specialized transgenic strains or extensive affinity purification protocols, and can be adapted for use to any mutant worm strain with reasonable numbers of immature germ cells. Thus, this technically straightforward and flexible procedure should greatly improve the accuracy and resolution of future genomic studies of germline gene regulation.

As we established conditions for the isolation procedure, we found that a balance must be reached in the number of Dounce strokes to achieve sufficient breakage and yet preserve the integrity of the nuclei. Thus, most animals remain intact throughout the process, and currently, the yield can vary from 15 to 30 nuclei per animal, depending on the condition of the Dounce homogenizers. We suspect that certain mutant strains that alter cuticle integrity, worm shape, or otherwise affect susceptibility to breakage might further improve efficiency and reduce the effort spent culturing worms.

The RNA-seq data clearly demonstrates that IGN display a strong enrichment for genes known to have germline-enriched expression. One important distinction between the IGN dataset and all other previously defined expression datasets is that IGN presumably have comparatively little contribution from cytoplasmic RNA pools; thus nuclear transcripts are likely better represented. This property allowed us to demonstrate that many genes expected to be expressed specifically in somatic cells actually exhibit some expression in germ nuclei (see Fig. 3f), and supports the many published observations [12–14] that cytoplasmic post-transcriptional mechanisms are necessary to dampen or block these transcripts. In the future, this feature should be useful for analysis of nascent transcription and of precursor transcripts, for instance, for profiling changes in pre-mRNAs in splicing or RNA-processing mutants.

Similarly, the ChIP-seq analysis of H3K27ac and H3K4me3 levels in IGN and somatic tissues (SOM) clearly demonstrates very distinct modification profiles. Genes known to be expressed specifically in germ cells exhibit the presence of these two activating modifications only in IGN, providing supporting molecular evidence that the IGN protocol results in purified germ nuclei. Somewhat surprisingly, we found a much more straightforward correlation between the presence of histone modifications and gene expression in IGN relative to SOM. More genes with IGN-enriched expression had one or both modifications than genes with SOM-enriched expression. Strikingly, genes with IGN-enriched expression retained levels of SOM-H3K4me3 that are actually higher than for genes with SOM-enriched expression (Fig. 5a and b). This observation perhaps explains why the number of X-linked genes with SOM-H3K4me3 is still below expectation (Fig. 4a), as genes with germline-enriched expression are generally not present on the X and yet make up a significant fraction of the SOM-H3K4me3 signal. Genes with IGN-enriched expression could exhibit persistent H3K4me3 in the soma because this gene set includes many “housekeeping” genes that function in fundamental cellular processes required by many cell types that are simply expressed at higher levels in IGN relative to SOM. Alternatively, this persistent H3K4me3 mark could be important for germline-dependent transgenerational epigenetic modulation of longevity [35], for example.

Conversely, the relatively poor association between activating histone modifications and gene expression in the soma could be due in part to the mixing of multiple cell types in the sample. Additionally, these analyses were conducted in adult somatic tissues, which have been developmentally stable for some time. Thus, transcript accumulation might be relatively uncoupled from genes currently marked as transcriptionally active by the presence of H3K27ac and/or H3K4me3 in upstream regulatory regions. Finally, a recent publication examining H3K4me3 during aging identified two types of patterns– one in which the modification covered gene bodies and another in which it was concentrated at promoters as expected [34]. Similarly, we detected H3K4me3 on both gene bodies and promoters (Additional file 8: Figure S8).

## Conclusions

In sum, these analyses show that even straightforward comparisons under wild type conditions can yield new insights when performed in isolated cell types. This newly developed procedure to isolate germ nuclei is simple and robust and should be readily employed and adapted to explore many different biological questions regarding gene regulatory and nuclear organization mechanisms in the *C. elegans* germ line.

## Methods

### *C. elegans* strains

Strains were maintained by standard methods unless otherwise indicated [36]. Whole-genome sequenced VC2010 (a substrain of N2) was used as the wild type strain. All worm culture was performed at 20 °C, except for *glp-1(q224)*, which was maintained at 15 °C and shifted to 25 °C after synchronized L1 s were hatched to induce sterility.

OP383 *unc-119(tm4063)* III; *wgIs383* [*oef-1*::TY1::EGFP::3x-FLAG + *unc-119*(+)] [37].

AZ212 *unc-119(ed3)*; *ruIs32* [*pie-1*p::GFP::H2B + *unc-119*(+)] III [18].

JK1107 *glp-1(q224)* III [19].

### Isolation of germline nuclei

Worms were grown to starvation on 15-cm NGM plates. Starved worms were washed with M9 and collected in a 15 mL conical tube. Worms were floated in 15 mL M9 for 5 min and the upper 6 mL worm solution containing mostly L1 s was transferred to a new 15 mL tube. 45 K L1 s were plated to each peptone enriched plate. Worms were grown to gravid on peptone enriched plates, bleached, and hatched overnight in M9 for 16–24 h. 50 K L1 s were plated to each enriched plate and grown until the young adult stage. Animal preparations for nuclei isolation were prepared at different scales as follows: worms from eighteen enriched plates (~ 1 million) were used per ChIP-qPCR or ChIP-seq experiment, and worms from six enriched plates (~3X10^5^) were used for RNA-seq. Young adult animals were harvested at various times after plating synchronized L1 s (VC2010: 54–56 h, OP383: 67–69 h, AZ212: 68–69 h), when most of the animals had 4–10 embryos. Worms from every six plates (~3X10^5^) were collected into one 50 mL conical tube and spun at 3100 rpm for 2 min and then washed 3x in M9.

For ChIP-qPCR and ChIP-seq, worms were crosslinked in 50 mL 2% formaldehyde for 30 min in three 50 mL conical tubes at room temperature [38]. Formaldehyde was quenched by 1 M Tris (pH 7.5) wash. Worms were then washed two more times with M9. Worms were then washed in the same tubes with 10 mL of prechilled Nuclei Purification Buffer (NPB; 50 mM HEPES pH = 7.5, 40 mL NaCl, 90 mM KCl, 2 mM EDTA, 0.5 mM EGTA, 0.2 mM DTT, 0.5 mM PMSF, 0.5 mM spermidine, 0.25 mM spermine, 0.1% tween 20, and cOmplete proteinase inhibitor cocktail (Roche) – 1 tablet per 25 mL NPB) [39, 40]. Worms from every six peptone enriched plates were resuspended with prechilled NPB to a final volume of 6 mL and subsequently transferred to a prechilled 7 mL glass Dounce homogenizer (Wheaton, Clearance: 0.05 +/− 0.025 mm). All subsequent steps were performed at 4 °C or on ice. One Dounce was used per set of 6 plates. A total of 15 loose and 22–30 tight strokes with a quarter turn after each stroke was performed to homogenize worms. After every 15 Dounce strokes, the sample was held for 5 minutes on ice. The optimal number of tight strokes depends on worm stage, worm genotype and the condition of the Dounce homogenizer. A fraction of broken worms between 8 and 15% after the last tight stoke normally related to good release of germline nuclei. NPB was then added to a final volume of 10 mL for worms from every 6 plates. The combined 30 mL worm solution from 3 Dounce sets were transferred to a prechilled 50 mL conical tube and vortexed on medium-high speed for 30 s, followed by 5 min on ice. The vortex and ice incubation steps are repeated one time to release more nuclei. The solution was passed through six 40 μm cell strainers (Fisherbrand) and six 20 μm cell strainers (pluriSelect) to remove worm debris. Two more 20 μm cell strainers were used for the filtrate to further remove worm debris. Isolated nuclei were collected by centrifugation at 3100 rpm for 6 min at 4 °C. The supernatant was removed and the nuclei were resuspended with 1 mL NPB. The nuclei were then transferred to a nonstick 1.5 mL tube (Ambion). A 5 μL aliquot of nuclei were stained with DAPI and counted with a hemacytometer (Hausser Scientific). Normally 15–30 million nuclei can be isolated from around 1 million young adult animals. The rest of the nuclei were pelleted at 4000 rpm for 5 min at 4 °C. Most of the supernatant was removed so that ~ 20 μL NPB was left. The nuclei were gently pipetted to mix and then flash frozen in liquid nitrogen and stored at − 80 °C until sonication.

For RNA-seq, worms from six peptone enriched plates were incubated for 30–45 min in M9 buffer with shaking after the third M9 wash to reduce intestinal bacteria. Worms were centrifuged at 3100 rpm for 2 min, then washed with 10 mL of NPB. Worms were then resuspended with prechilled NPB (with 3 μL/mL RNase Inhibitor (Invitrogen) hereafter) to a final volume of 6 mL. All of the Dounce and following steps were performed as for ChIP-seq except for the number of tight strokes (15–22), and two 40 μm cell strainers and two 20 μm cell strainers were used to filter worm debris. Finally, 500 μL TRIzol (Invitrogen) was added to the pelleted nuclei, and then flash frozen in liquid nitrogen and stored at − 80 °C until RNA isolation.

### Immunostaining

GFP immunostaining was performed on isolated nuclei from OP383, AZ212 and wild type VC2010 worms from six peptone enriched plates for each genotype. Nuclei were isolated as described for ChIP-qPCR and ChIP-seq, except that nuclei were not flash frozen after isolation. One-third of the nuclei was used for an individual immunostaining experiment. All subsequent washes and incubations were performed in 1.5 mL tubes with rotation. 1 mL − 20 °C methanol was added to isolated nuclei for additional fixation for at least 30 min at 4 °C. Nuclei were spun at 4000 rpm for 5 min and washed 3 times with 1 mL PBST (PBS with 0.1% Tween 20) for 5 min per wash at room temperature. Nuclei were blocked with 1 mL 0.5% BSA (American Bioanalytical) in PBST for 30 min. Then 120 μL of 1:2000 anti-GFP (ab13970, Abcam) diluted in blocking solution was added and incubated overnight at 4 °C. Nuclei were spun at 4000 rpm for 5 min at room temperature. Supernatant was removed and 1 mL 0.5 μg/ml DAPI in PBST was added to stain the nuclei for 10 min. Nuclei were washed 2 times with PBST for 5 min for each wash. 120 μL 1:500 goat-anti-chicken Alex Fluor 488 (A-11039, Invitrogen) secondary antibody in PBST was used to incubate the nuclei overnight at 4 °C. Nuclei were washed 3 times with 1 mL PBST for 10 min. Nuclei were spun at 4000 rpm for 5 min and supernatant was removed. Nuclei were resuspended with 15 μL PBST by gently pipetting. 15 μL antifade mounting medium (Vectashield) was added to the nuclei. 15 μL nuclei were placed on agarose pads under a cover glass with tiny dots of Vaseline on four corners. Images were obtained with a Zeiss Axioplan microscope with a 100X objective and processed with AxioVision software.

### RNA-sequencing

RNA isolation was performed on VC2010 IGN and *glp-1(q224)*, with two biological replicates for each sample*.* VC2010 germline nuclei were isolated as described for isolation of germline nuclei.

*glp-1(q224)* animals were cultured to starvation on 15-cm NGM plates at 15 °C. L1 worms were floated and 10 K L1 s were plated to one 15-cm NGM plate. Worms were cultured at 15 °C for four days until gravid. Adult worms were bleached and embryos were incubated with shaking at 15 °C for 36–42 h. 15 K L1 s were plated to one peptone enriched plate and cultured at 25 °C for 46–48 h until adult stage. Worms were harvested by washing with M9 3x and were incubated with shaking for 30–45 min in M9 buffer. Worms were centifuged at 3100 rpm for 2 min. Supernatant was removed and 500 μL TRIzol was added to the worm pellet. Worms were frozen in liquid nitrogen and stored at − 80 °C until RNA isolation.

Total RNA isolation was performed with standard TRIzol RNA extraction. Approximately 3 μg RNA is typically obtained from each IGN sample collected from six enriched plates. Total RNA was then treated with DNA-*free* rDNase I (Ambion) and cleaned up using RNeasy Mini Kit (Qiagen). rRNA was depleted by Ribo-Zero rRNA Removal Kit (Illumina). The Yale Center for Genome Analysis (YCGA) prepared libraries for each sample using Kapa Biosystems reagents. At least 20 million 75-bp paired-end reads were acquired for each library using Illumina HiSeq2500.

### RNA-seq analysis

The raw paired-end RNA-seq fastq reads were first mapped to rRNA build by Bowtie2 (v2.1.0) [41], then the remaining unmapped reads were further aligned to ce10 genome by HISAT2 (v2.0.4) [20] with the mode suppressing the unpaired reads. The gene annotation was downloaded from UCSC Genome Browser, filtered to remove transcripts < 50 nt. The expression level of FPKM and significant status were determined by Cuffdiff (v2.2.1) [21]. The bigwig files were generated by SAMtools (v1.3) [42] and BEDtools (v2.17.0) [43], and then normalized to 10 million mapped reads for visualization in Genome Browser. As a secondary analysis, the number of read pairs for each gene were counted by FeatureCounts (v1.5.2) [44], then subjected to DESeq2 (1.22.2; R Bioconductor) package for further analysis. Adjusted *p* value was set at 0.01 for the significance level. The MA plot and PCA plot in Additional file 3: Figure S3 were generated accordingly.

The intron intervals were extracted from ce10 genome annotation. FeatureCounts (v1.5.2) were employed to count the number of reads that overlapped with intron region of each gene. Genes with more than 10 intronic reads across all the samples were kept for further analysis. The read counts were then normalized to 10 million mapped reads to allow for comparisons.

### Preparation of VC2010 IGN and *glp-1(q224)* chromatin and ChIP-sequencing

ChIP-seq of VC2010 IGN was performed using a combined protocol [45, 46] and ChIP-seq on *glp-1(q224)* adult animals was performed as previously reported [46]. Two replicates of ChIP-seq samples were processed for both VC2010 IGN and *glp-1(q224).* VC2010 IGN samples were acquired as described above. 120 μL Nuclear Lysis Buffer (NLB, 50 mM Tris pH = 8, 10 mM EDTA, 1% SDS, 0.5 mM PMSF, 2X cOmplete proteinase inhibitor cocktail) was added to each IGN sample. IGN samples were vortexed vigorously for 1 min and left on ice for 1 min. The vortex step was repeated. IGN samples were sonicated at 2 °C in a water bath sonicator (Misonix S-4000). 20% amplitude and 10 s on/10 s off pulses were used for a total processing time of 20 min, resulting in enrichment for 100–650 bp DNA fragments. 1.2 mL prechilled FA buffer (50 mM HEPES pH 7.5, 1 mM EDTA, 1% Triton X-100, 0.1% sodium deoxycholate, 150 mM NaCl, add before use: 1 mM DTT, 0.5 mM PMSF, cOmplete proteinase inhibitor cocktail – 1 tablet per 25 mL NPB) was added per IGN sample. 1:20 volume of 20% Sarkosyl solution was added. Sonicated samples were spun at 13,000 g for 5 min at 4 °C. The supernatant was transferred to a new nonstick 1.5 mL tube. 5% of lysate (70 μL) was removed for the input sample and stored at − 20 °C until the following day to prepare input DNA. 5 μg of anti-H3K27ac (39,685, Active Motif) or anti-H3K4me3 (61,379, Active Motif) was incubated with each IGN sample overnight at 4 °C with rotation.

*glp-1(q224)* animals were cultured as described in RNA-sequencing, except that 50,000 L1 s were plated on each peptone enriched plate. Adult *glp-1(q224)* animals from three peptone enriched plates (~ 1.5X10^5^) were harvested by 3 washes with M9. Worms were crosslinked in 50 mL 2% formaldehyde for 30 min in a 50 mL conical tube at room temperature. Formaldehyde was quenched by 1 M Tris (pH 7.5) wash. Worms were then washed 2 more times with M9. Worms were transferred to a 15 mL conical tube and washed with 15 mL prechilled FA buffer. Worms were spun at 3100 rpm for 2 min. All but ~ 200 μL FA buffer was removed and worms were frozen in liquid nitrogen and stored at − 80 °C. Worm pellets were thawed on ice and 750 μl of FA buffer was added to each sample. Samples were transferred to a 2 mL Kontes Dounce (Kimble Chase). Samples were Dounced 15 times with the small “A” pestle for two cycles with a 1 minute hold on ice between each cycle. Samples were then Dounced 15 times with the large “B” pestle for four rounds with a 1 minute hold in between. Samples were transferred to a 15 mL conical tube and FA buffer was added to a final 1.5 mL volume. A quick spin was performed to collect the sample. Samples were sonicated with a SFX250 sonifier (Branson) in an ice bath at 22% amplitude with 10 s on/1 min off pulses for 34 cycles in a total process time of 5 min and 40 s. 100–650 bp DNA fragments were enriched after sonication. The sample was transferred to a nonstick 2 mL tube (Ambion) and spun at 13,000 g for 15 min at 4 °C. Supernatant was transferred to a new nonstick 2 mL tube. The protein concentration of the lysate was determined by Bradford assay, and a total of 4.4 mg protein was used for each ChIP sample. Prechilled FA buffer was added to each ChIP sample to bring the volume to 400 μL. 1:20 volume of 20% Sarkosyl solution was added to each ChIP sample. Samples were spun at 13,000 g for 5 min at 4 °C. The supernatant lysate was transferred to a new nonstick 1.5 mL tube. 5% of lysate (20 μL) was removed for input sample and stocked at − 20 °C overnight. 5 μg of anti-H3K27ac (39,685, Active Motif) or anti-H3K4me3 (61,379, Active Motif) was incubated with *glp-1(q224)* sample overnight at 4 °C with rotation. Both the VC2010 IGN and *glp-1(q224)* samples were treated the same hereafter.

The input samples were thawed the next day and 2 μL 10 mg/mL RNase A (Qiagen) was added to digest the input samples for 2 h at room temperature. 40 μL (~ 20 μL of actual beads) protein G Sepharose beads (GE Healthcare) were used for each ChIP sample and washed 4 times with 1 mL prechilled FA buffer. The beads were collected with at 2500 g for 2 min. The entire ChIP sample was transferred to the 1.5 mL tubes with pre-washed beads and rotated at 4 °C for 2 h. Elution buffer (1% SDS in TE, 250 mM NaCl) was added to input samples to bring volume up to 300 μL after RNase A treatment. 2.05 μL of 19.5 mg/mL Proteinase K (Roche) was added to input samples. Input samples were incubated at 55 °C for 3 h. The ChIP samples with beads were washed at room temperature by adding 1 mL of each of the following buffers and incubated for the specified time on a rotator: 2 times FA buffer for 5 min; 1 time FA-500 mM NaCl (50 mM HEPES pH 7.5, 1 mM EDTA, 1% Triton X-100, 0.1% sodium deoxycholate, 500 mM NaCl) for 10 min; 1 time TEL buffer (0.25 M LiCl, 1% NP40, 1% sodium deoxycholate, 1 mM EDTA, 10 mM Tris pH = 8.0) for 10 min; 2 times TE for 5 min. 150 μL elution buffer was added to each ChIP sample and placed in a 65 °C heat block for 15 min with a brief vortex every 5 min. The beads were spun down at 2500 g for 2 min and the supernatant transferred to new nonstick 1.5 mL tubes. The elution step was repeated and the supernatants were combined. 1.03 μL of 19.5 mg/mL Proteinase K (Roche) was added to each ChIP sample and incubated at 55 °C for 1 h. All input and ChIP samples were transferred to 65 °C overnight to reverse crosslinks after Proteinase K treatment. The input and ChIP DNA was purified with a PCR purification kit (Qiagen) following the manufacturer’s protocol. 40 μL TE pH = 8 was used to elute DNA.

The Yale Center for Genome Analysis (YCGA) prepared the library and performed sequencing. The KAPA Hyper Library Preparation kit (KAPA Biosystems) was used for ChIP-sequencing library prep. DNA fragment ends were repaired with T4 DNA Polymerase, and Polynucleotide Kinase and “A” base added using Klenow fragment in a single reaction followed by ligation of custom adapters (IDT) using T4 ligase. Adaptor-ligated DNA fragments were purified and size selected with Agencourt AMPure XP magnetic beads (Beckman Coulter). Adaptor-ligated DNA fragments were amplified by LM-PCR using custom-made primers (IDT). During LM-PCR, unique 10 base indices were inserted at each DNA fragment and amplified products were purified. The prepped samples were then loaded on to a single-end flow cell and subjected to sequencing. 10–25 million 75-bp single-end reads were acquired for each library using Illumina HiSeq2500 rapid run mode.

### ChIP-seq analysis

The raw ChIP and corresponding input fastq sequencing reads were mapped to the genome (version ce10) by Bowtie2 (v2.3.2) with default parameters. The datasets for two replicates were merged for further analysis. To eliminate the replicate bias, the alignment file (bam) from the sample with larger library size was downsampled to the size of the replicate with smaller library size, and then merged together by Samtools (v1.3). Peaks were called by MACS2 (v2.1.1) [47] with the key parameter (−q 0.001 --nomodel --extsize 150). Wig files were generated by a custom script (https://github.com/guifengwei/glib/blob/master/bam2wig.py).

Metagene analysis custom scripts were used for extracting the value from certain genic regions and averaged for metagene profile by DANPOS [48] with the key parameters (−-genomic_sites TSS --flank_up 2000 --flank_dn 2000 --bin_size 50 –exclude P 0.001). Random transcripts were selected for genes with multiple transcription start sites (TSSs). Genes in categories (1895 ubiquitous genes, 2230 germline-enriched genes, 169 germline-specific genes, 1181 soma-specific genes and 858 sperm genes) for metagene profile analysis (Fig. 4b and c) were described previously [13].

Target calling analysis was performed using ce10 annotation. For each H3K27ac peak summit, the four closest features were identified, regardless of strand, within each of the following categories: coding mRNA, non-coding RNA, and 21uRNA. In addition, the closest feature overall was labeled along with the distance to the peak summit and whether it was closest to the 5′ or the 3′ end of the feature and whether it was upstream or downstream of that 5′ or 3′ end. For each H3K4me3 peak summit, all features that fell anywhere within the region from the start to the end of the peak were listed. For this study, only coding targets were selected for further analysis. Several parameters were used to determine the representative peak for each target gene to which more than one peak was assigned. For H3K27ac ChIP-seq: 1. All peaks with binding at the 3′ end were removed; 2. *The* peak with the highest ChIP enrichment value was selected if all peaks assigned to the same target were within 1 kb distance of the TSS; 3. If the highest peak was within 1000–1200 bp, and the ChIP enrichment value is more than 2 times the highest peak within 1 kb, then it was retained. *For H3K4me3 ChIP-seq:* the peak with the highest ChIP enrichment value was selected. The unique ChIP peak enrichment value was then determined for each target gene *(*Additional files 12, 13, 14, and 15*).*

H3K27ac and H3K4me3 peaks across chromosomes relative to chromosome length were calculated for IGN- or SOM-H3K27ac ChIP-seq and IGN- or SOM-H3K4me3 ChIP-seq datasets. Significance was determined using a Pearsons Chi Square.

H3K4me3 heatmaps were generated by ngs.plot.r (v2.63) [49] with the default parameters except (−G ce10 -R genebody -SC Gobal). The genes used to plot heatmaps were the IGN or SOM-enriched transcripts identified by Cuffidiff.

### ChIP-qPCR

Samples were prepared as for ChIP-seq, except samples were eluted in 50 uL water. 1 μL of input or ChIP DNA from H3K27ac ChIP experiments, 300 nM of primer, and 12.5 μL FastStart Universal SYBR Green Master (Rox) (Roche) was used in a 25 μL quantitative PCR reaction. ChIP-qPCR were performed as previously described [50]. Relative fold enrichment of germline genes was normalized to negative control *elt-2*. Primers were determined by utilizing ChIP peaks previously identified for L3 N2-H3K27ac ChIP-chip data from the ENCODE project (GSM624432 and GSM624433) [27] or promoter regions with relatively similar distance from the start codon (for negative control).

List of ChIP-qPCR primers.

**Table:** 

Primer	Primer Sequence (5′-3′)
*him-3* F1	ACAATTTCTCAGCAGCAGCA
*him-3* R1	GGCATGGACGTTTGTCTTCT
*him-3* F2	CATTCCGAGCTTCTTGTCGT
*him-3* R2	GTCCGAAATTTGATGCTGCT
*him-3* F3	TCTCGCTTGTTAGCCTCCAT
*him-3* R3	TCGATCTCGTCCCAATTTTC
*oef-1* F1	GCATGTTGCGAAACTGAGAA
*oef-1* R1	CACATTGCCCATACAGCAAG
*oef-1* F2	GCACCAACTGGAAACTTGCT
*oef-1* R2	TCGCTTCTCATTTCATGCAC
*oef-1* F3	TCTCGCTTGTTAGCCTCCAT
*oef-1* R3	TCGATCTCGTCCCAATTTTC
*C37H5.15* (positive control) F	CCGATAACATGTCCCTTTGG
*C37H5.15* (positive control) R	CTTTCCGCACGATCATTCTT
*elt-2* (negative control) F	CTGGAAGTGGGTGGTTGTCT
*elt-2* (negative control) R	GGCACAAAGCGTATTGGTTT
*myo-3* F	CCCAGTTACATTCCCCACTG
*myo-3* R	TCCTTCGTTTTCCGATGAAC

## Additional files

Additional file 1: **Figure S1** Isolated germline nuclei from a second germline transgenic strain. (A) Young adult *pie-1*p::GFP::H2B transgenic worm showing germline expression. GFP::H2B is expressed in the nuclei of germ cells at all developmental stages, including oocytes and embryos. (B) Isolated nuclei from *pie-1*p::GFP::H2B young adult worms were immunostained with GFP (green) and stained with DAPI (blue). Nuclei stained with both GFP and DAPI (89.1%, *n* = 2111) were considered germline nuclei. Two independent experiments were performed. (C) Isolated nuclei from wild type VC2010 N2 young adult worms were immunostained with GFP (green) and stained with DAPI (Blue). Scale bars, 20 μm in (A), 5 μm in (B) and (C). (PDF 6536 kb)

Additional file 2: **Figure S2** A small fraction of sperm are present in total isolated germline nuclei. Isolated total nuclei from young adult *pie-1*p::GFP::H2B transgenic worms. Nuclei were immunostained with GFP (green) and stained with DAPI (Blue). Arrows indicate two non-GFP stained nuclei with characteristics of sperm. Scale bar, 5 μm. (PDF 4083 kb)

Additional file 3: **Figure S3** DEseq2 analysis of gene expression levels in SOM and IGN. (A) MA plot showing the differential gene expression pattern for SOM and IGN samples. The differentially expressed genes between SOM and IGN were represented with red dots (p adj < 0.01). Y-axis: M (log_2_TPM (fold change)). X-axis: A (Mean of normalized counts). (B) PCA analysis indicating good correlation between replicates for SOM and IGN samples. (C) Venn diagrams displaying the overlap between Cuffdiff-called and DESeq2-called differentially expressed genes in SOM (top) and IGN (bottom) samples. Of the Cuffdiff-called differentially expressed genes in SOM or IGN, 91.48% or 86.42% were also called by DESeq2, respectively. (D) Boxplot exhibiting the significance of differentially expressed gene sets either called by both Cuffdiff and DESeq2 or DESeq2 only. (E) Boxplot displaying the overall gene expression (TPM) for tissue-enriched transcripts identified by Cuffdiff. Transcripts with less than 10 total read counts across all the samples were removed for TPM analysis. Thus, 3941 out of 3965 SOM-enriched transcripts (blue) or 4940 out of 5075 IGN-enriched transcripts (red) in either SOM RNA-seq or IGN RNA-seq were analyzed. Each box indicates the median and interquartile range of TPM level. (PDF 941 kb)

Additional file 4: **Figure S4** Representative RNA-seq profiles and transcript abundance of IGN and SOM. (A-B) Genome browser views of representative RNA-seq profiles of wild type IGN (red) and SOM (blue) on genomic regions from chromosome IV (A) and I (B), showing tissue-specific transcript abundance. The green box highlights an example of primary or partially processed transcripts of *suco-1* in IGN. Black arrows indicate signal detected in an intron in IGN (B). (C) A boxplot displaying the overall abundance and distribution of gene expression levels (FPKM) for 3965 SOM-enriched transcripts (blue) and 5075 IGN-enriched transcripts (red) in either IGN RNA-seq or SOM RNA-seq. Each box indicates the median and interquartile range of FPKM level. (PDF 1247 kb)

Additional file 5: **Figure S5** GO analysis of genes present exclusively in the dissected gonad dataset. Eighteen of the most significant Gene Ontology Biological Process terms for the 6606 transcripts present exclusively in the dissected gonad [23] when compared to the IGN dataset in Fig. 3e. (PDF 862 kb)

Additional file 6: **Figure S6** Analysis of Intron reads for IGN RNA-seq and SOM RNA-seq. (A) The percentage of intron reads (reads that overlap with introns or fully located in introns) for indicated RNA-seq samples. (B) Smoothed scatter plot showing the TP10M value of intron reads for each gene in IGN and SOM samples. TP10M values were calculated by the sum of intronic reads for each gene, then normalized to 10 million mapped reads. (PDF 1629 kb)

Additional file 7: **Figure S7** Confirmation of germline-enriched H3K27ac modification in isolated germ nuclei by ChIP-qPCR. Two previously characterized germline-expressed (*him-3* and *oef-1*) and soma-expressed genes (*elt-2* and *myo-3*) were examined for abundance of H3K27ac in IGN by ChIP-qPCR. Three sets of primers were tested for each germline-specific gene. *C37H5.15* served as positive control. *Elt-2* served as negative control and was used to calculate fold enrichment. ChIP results are expressed as percent of input using Ct values (A) and fold enrichment of H3K27ac modification normalized to *elt-2* (B). (PDF 1136 kb)

Additional file 8: **Figure S8** Heatmap of H3K4me3 levels for genes with tissue-enriched expression. Heatmap displaying the levels of H3K4me3 for genes with SOM-enriched expression or IGN-enriched expression, as assayed by IGN H3K4me3 ChIP-seq and SOM H3K4me3 ChIP-seq. (PDF 3310 kb)

Additional file 9: **Table S1** Quantification of fraction of germline nuclei (DOCX 14 kb)

Additional file 10: IGN and soma RNA-seq expression analysis (XLSX 4868 kb)

Additional file 11: IGN transcripts compared to other datasests (XLSX 3951 kb)

Additional file 12: IGN_H3K27ac_q0.001_peaks_ce10 (XLSX 5606 kb)

Additional file 13: SOM_H3K27ac_q0.001_peaks_ce10 (XLSX 5600 kb)

Additional file 14: IGN_H3K4me3_q0.001_peaks_ce10 (XLSX 3928 kb)

Additional file 15: SOM_H3K4me3_q0.001_peaks_ce10 (XLSX 4603 kb)

Additional file 16: SOM-CHIP for IGN-enriched expression (XLSX 838 kb)

Additional file 17: IGN-CHIP for SOM-enriched expression (XLSX 737 kb)

## Data Availability

The datasets supporting the conclusions of this article are available in Gene Expression Omnibus database under accession GSE117061.

## Abbreviations

ChIP-seq: Chromatin immunoprecipitation followed by high throughput sequencing
DAPI: 4′,6-diamidino-2-phenylindole
FPKM: Fragments Per Kilobase of transcript per Million mapped reads
IGN: Isolated germ nuclei
RNA-seq: RNA isolation followed by high throughput sequencing
SOM: Somatic tissues
