# Supplementary figures and images for: Isolated C. elegans germ nuclei exhibit distinct genomic profiles of histone modification and gene expression
Source: BMC Genomics. 2019 Jun 17;20:500. doi: 10.1186/s12864-019-5893-9 (PMC6580472; doi:10.1186/s12864-019-5893-9)

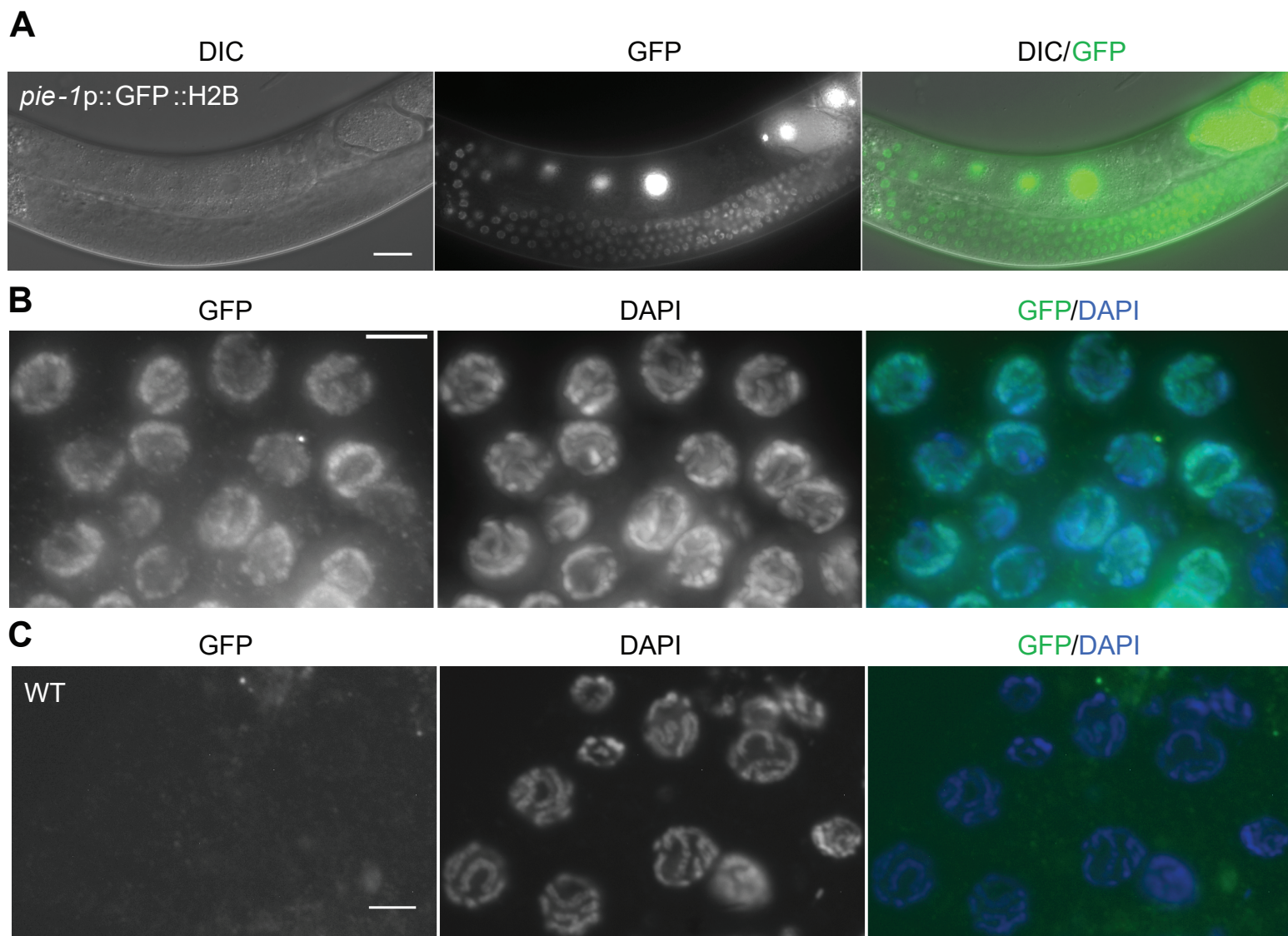

Figure S1

Supplement: Supplementary file 1 — Figure S1 Isolated germline nuclei from a second germline transgenic strain. (A) Young adult pie-1p::GFP::H2B transgenic worm showing germline expression. GFP::H2B is expressed in the nuclei of germ cells at all developmental stages, including oocytes and embryos. (B) Isolated nuclei from pie-1p::GFP::H2B young adult worms were immunostained with GFP (green) and stained with DAPI (blue). Nuclei stained with both GFP and DAPI (89.1%, n = 2111) were considered germline nuclei. Two independent experiments were performed. (C) Isolated nuclei from wild type VC2010 N2 young adult worms were immunostained with GFP (green) and stained with DAPI (Blue). Scale bars, 20 μm in (A), 5 μm in (B) and (C). (PDF 6536 kb) [file 12864_2019_5893_MOESM1_ESM.pdf]

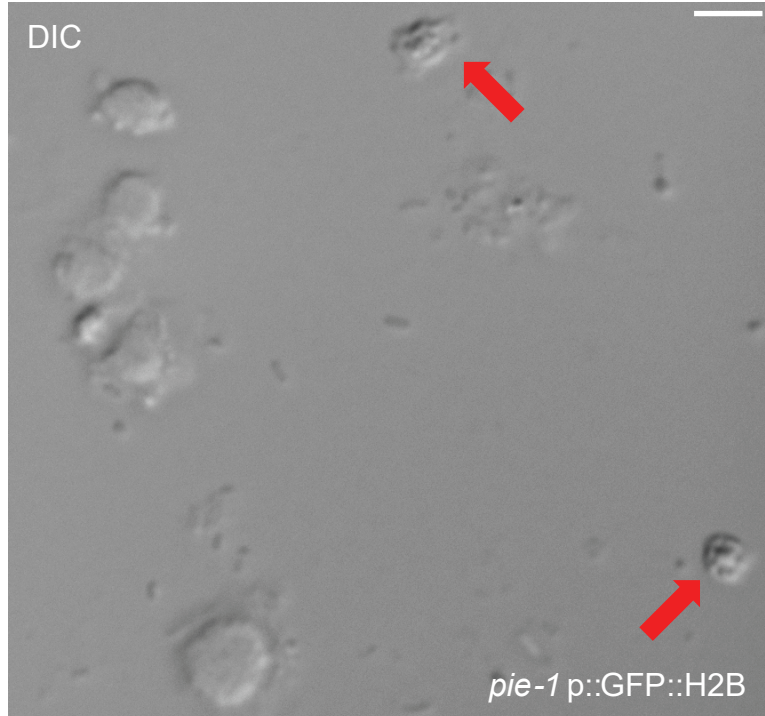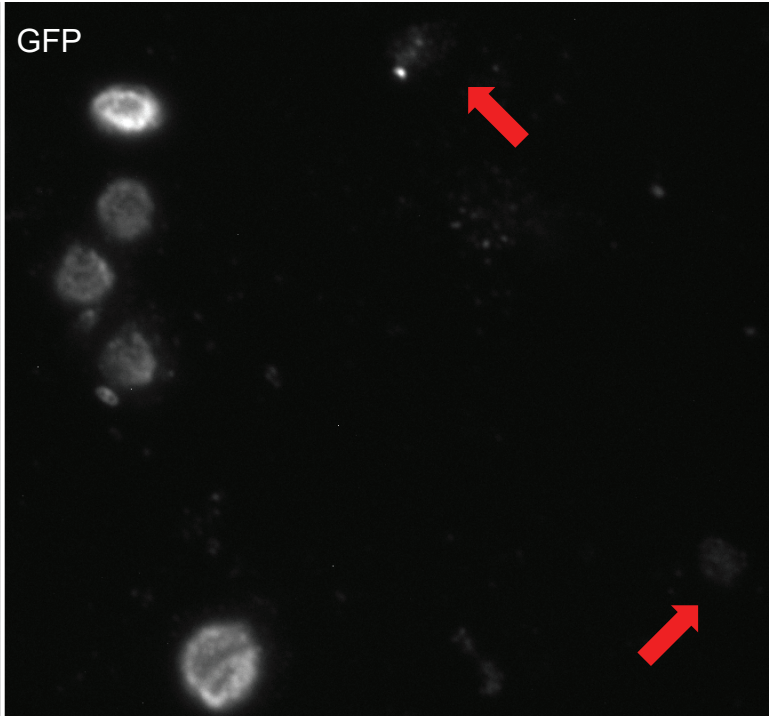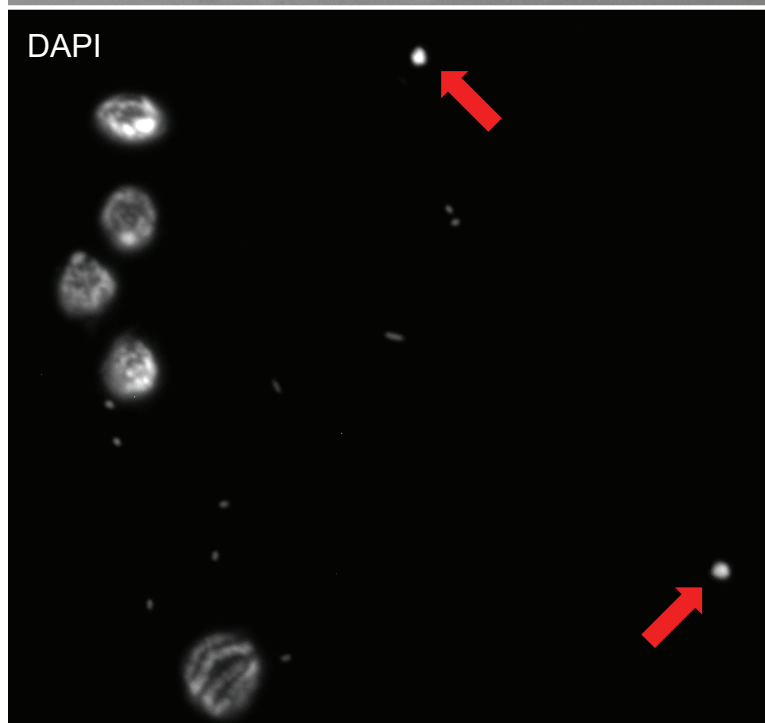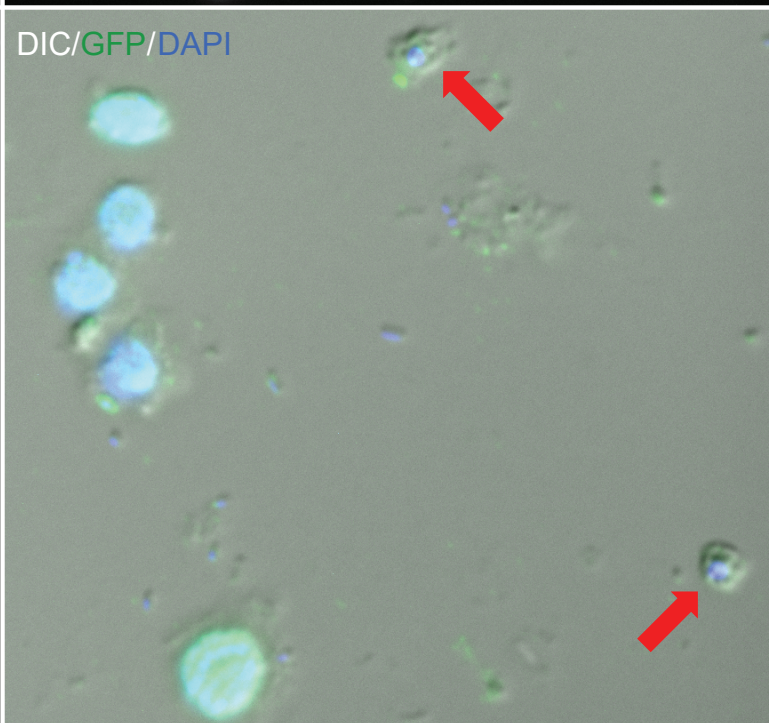

Figure S2

Supplement: Supplementary file 2 — Figure S2 A small fraction of sperm are present in total isolated germline nuclei. Isolated total nuclei from young adult pie-1p::GFP::H2B transgenic worms. Nuclei were immunostained with GFP (green) and stained with DAPI (Blue). Arrows indicate two non-GFP stained nuclei with characteristics of sperm. Scale bar, 5 μm. (PDF 4083 kb) [file 12864_2019_5893_MOESM2_ESM.pdf]

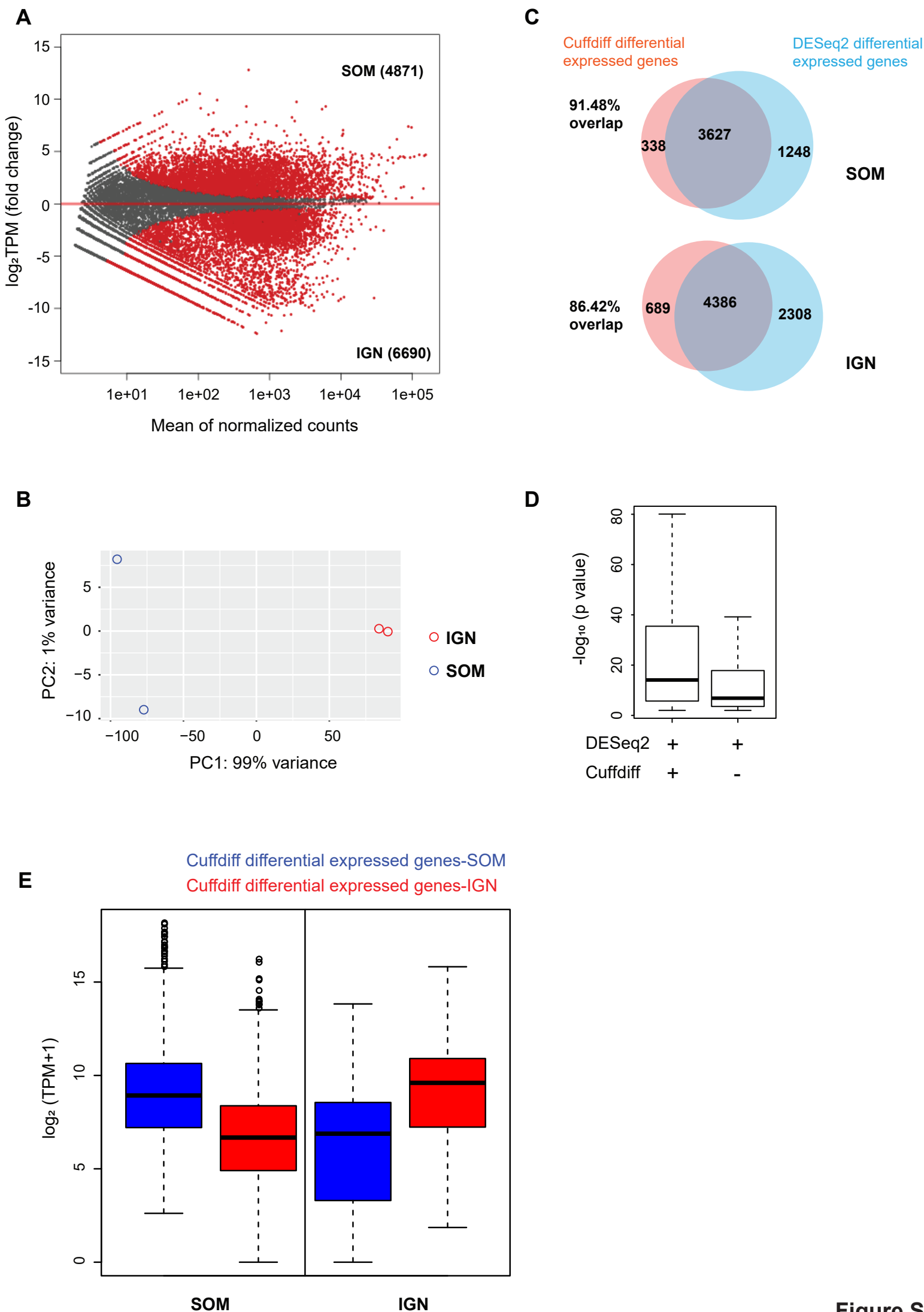

Figure S3

Supplement: Supplementary file 3 — Figure S3 DEseq2 analysis of gene expression levels in SOM and IGN. (A) MA plot showing the differential gene expression pattern for SOM and IGN samples. The differentially expressed genes between SOM and IGN were represented with red dots (p adj < 0.01). Y-axis: M (log2TPM (fold change)). X-axis: A (Mean of normalized counts). (B) PCA analysis indicating good correlation between replicates for SOM and IGN samples. (C) Venn diagrams displaying the overlap between Cuffdiff-called and DESeq2-called differentially expressed genes in SOM (top) and IGN (bottom) samples. Of the Cuffdiff-called differentially expressed genes in SOM or IGN, 91.48% or 86.42% were also called by DESeq2, respectively. (D) Boxplot exhibiting the significance of differentially expressed gene sets either called by both Cuffdiff and DESeq2 or DESeq2 only. (E) Boxplot displaying the overall gene expression (TPM) for tissue-enriched transcripts identified by Cuffdiff. Transcripts with less than 10 total read counts across all the samples were removed for TPM analysis. Thus, 3941 out of 3965 SOM-enriched transcripts (blue) or 4940 out of 5075 IGN-enriched transcripts (red) in either SOM RNA-seq or IGN RNA-seq were analyzed. Each box indicates the median and interquartile range of TPM level. (PDF 941 kb) [file 12864_2019_5893_MOESM3_ESM.pdf]

# A

## ChrIV

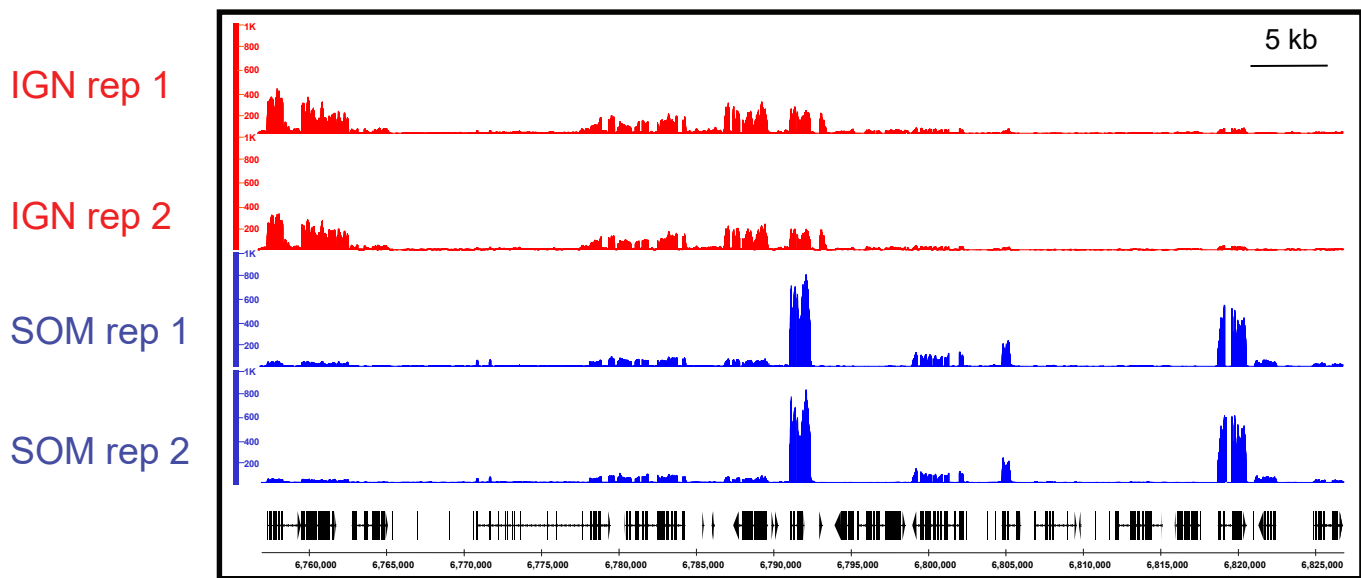

# B

## ChrI

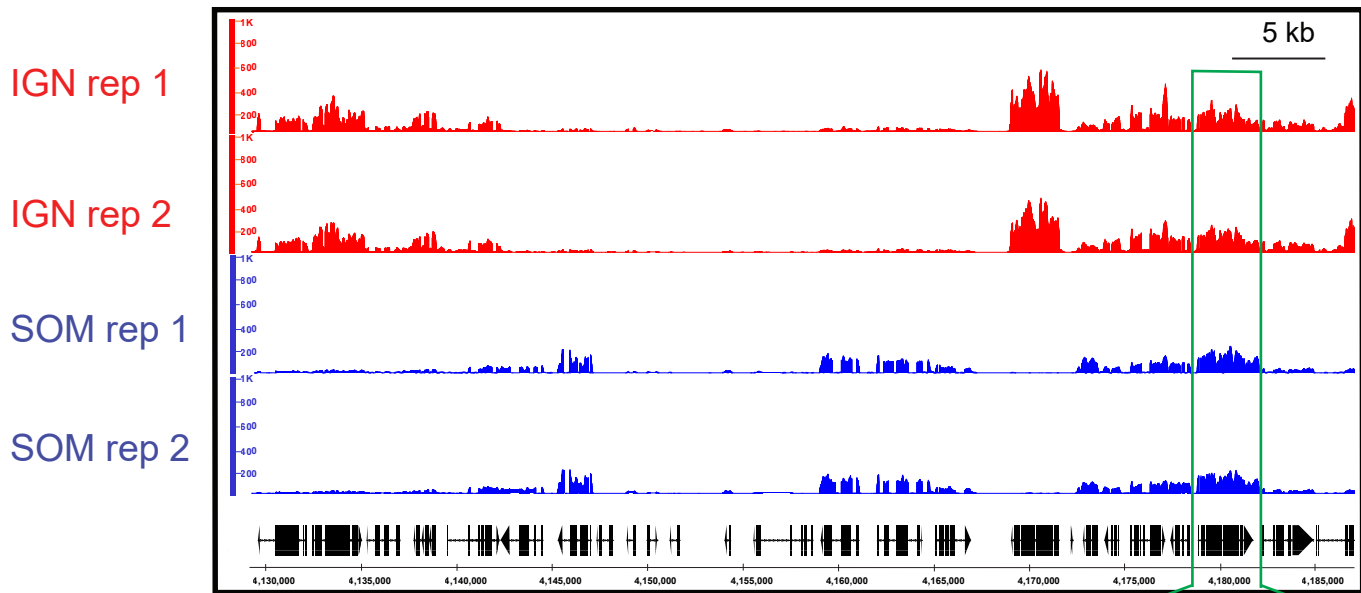

# C

SOM-enriched transcripts n=3965  
IGN-enriched transcripts n=5075

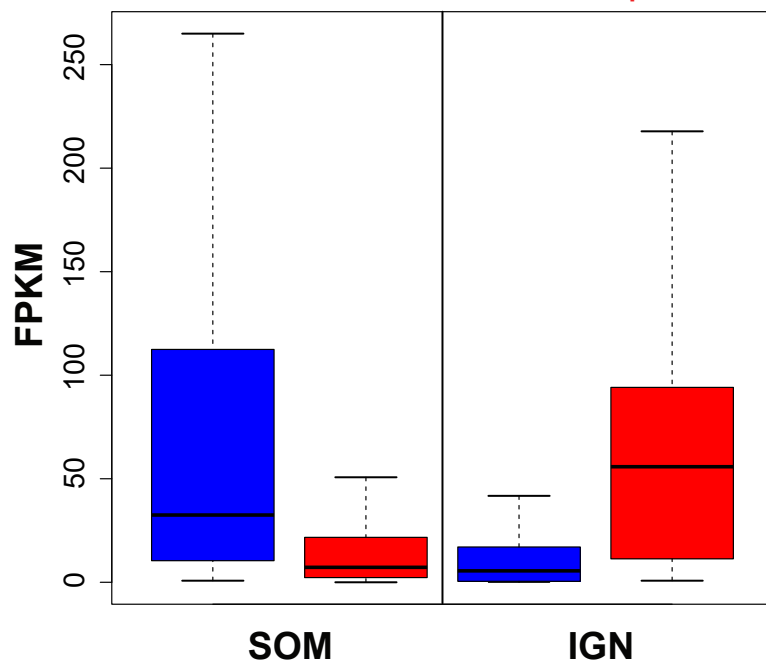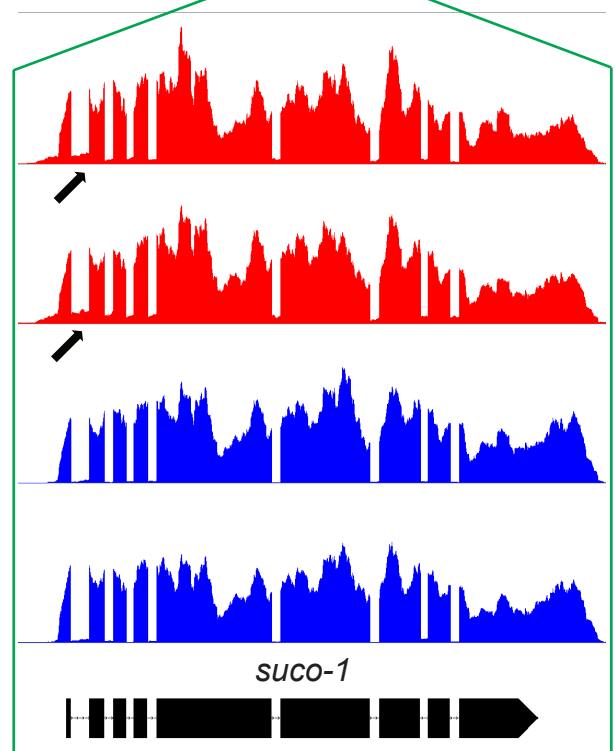

Figure S4

Supplement: Supplementary file 4 — Figure S4 Representative RNA-seq profiles and transcript abundance of IGN and SOM. (A-B) Genome browser views of representative RNA-seq profiles of wild type IGN (red) and SOM (blue) on genomic regions from chromosome IV (A) and I (B), showing tissue-specific transcript abundance. The green box highlights an example of primary or partially processed transcripts of suco-1 in IGN. Black arrows indicate signal detected in an intron in IGN (B). (C) A boxplot displaying the overall abundance and distribution of gene expression levels (FPKM) for 3965 SOM-enriched transcripts (blue) and 5075 IGN-enriched transcripts (red) in either IGN RNA-seq or SOM RNA-seq. Each box indicates the median and interquartile range of FPKM level. (PDF 1247 kb) [file 12864_2019_5893_MOESM4_ESM.pdf]

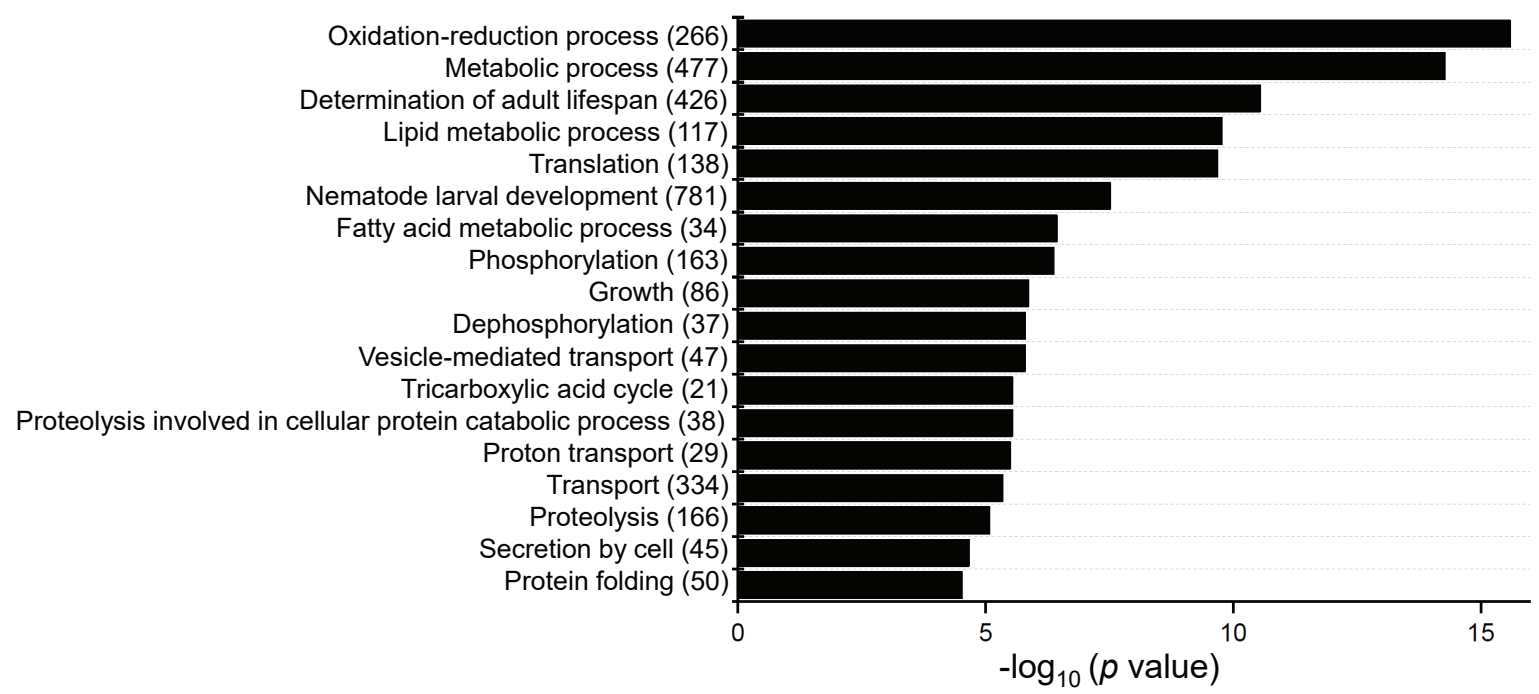

Figure S5

Supplement: Supplementary file 5 — Figure S5 GO analysis of genes present exclusively in the dissected gonad dataset. Eighteen of the most significant Gene Ontology Biological Process terms for the 6606 transcripts present exclusively in the dissected gonad [23] when compared to the IGN dataset in Fig. 3e. (PDF 862 kb) [file 12864_2019_5893_MOESM5_ESM.pdf]

**A**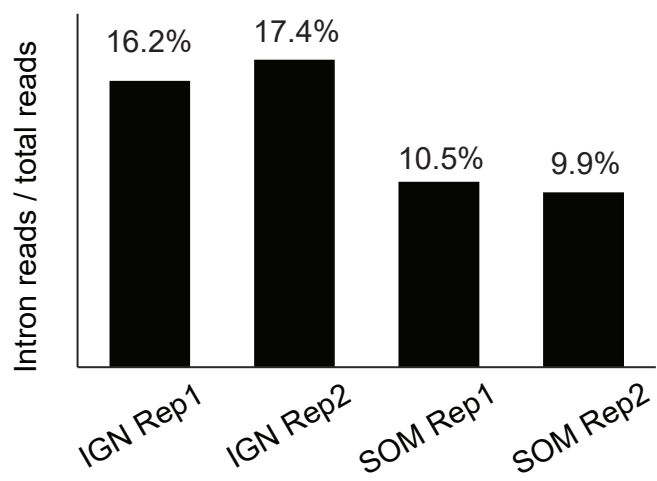**B**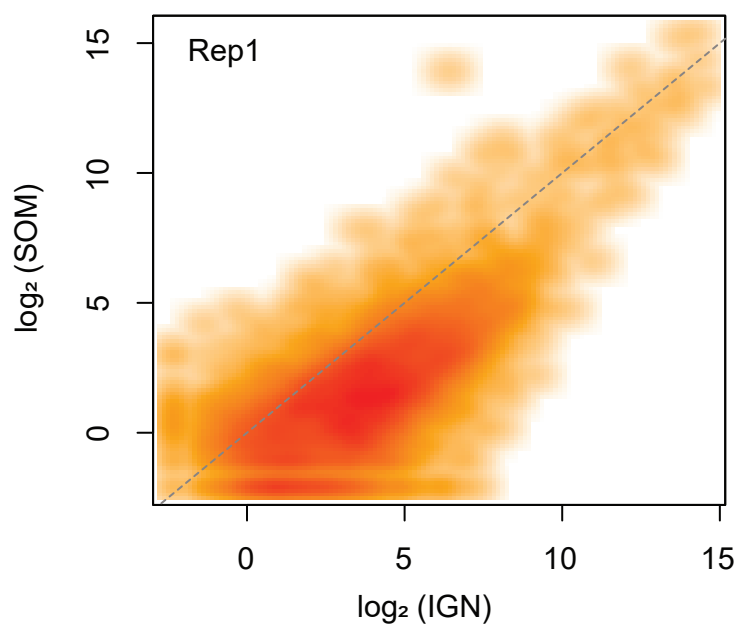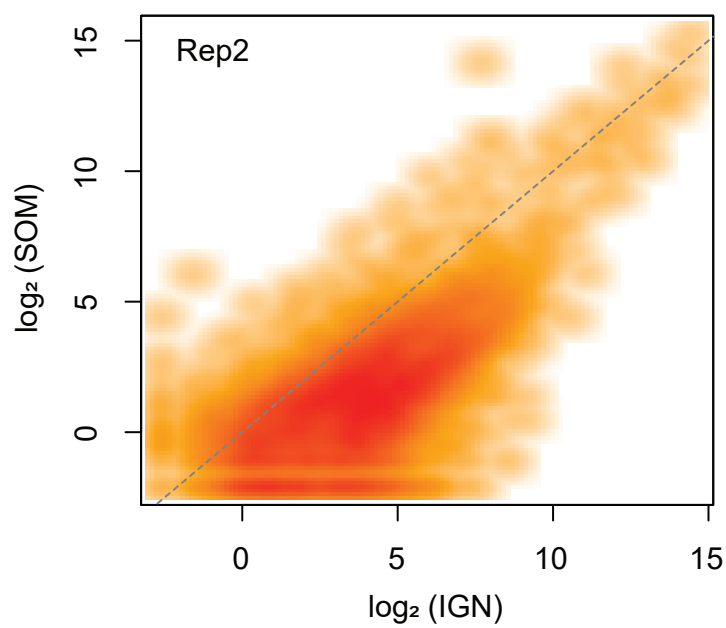

Supplement: Supplementary file 6 — Figure S6 Analysis of Intron reads for IGN RNA-seq and SOM RNA-seq. (A) The percentage of intron reads (reads that overlap with introns or fully located in introns) for indicated RNA-seq samples. (B) Smoothed scatter plot showing the TP10M value of intron reads for each gene in IGN and SOM samples. TP10M values were calculated by the sum of intronic reads for each gene, then normalized to 10 million mapped reads. (PDF 1629 kb) [file 12864_2019_5893_MOESM6_ESM.pdf]

**A**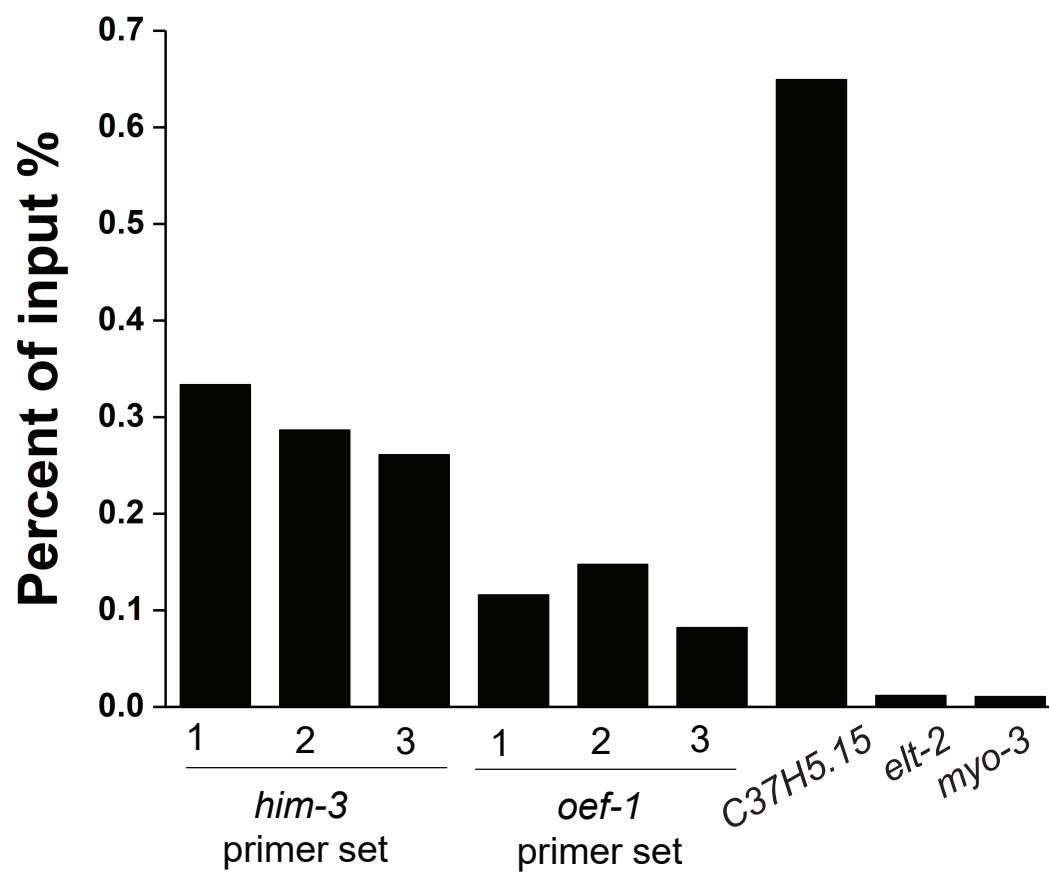**B**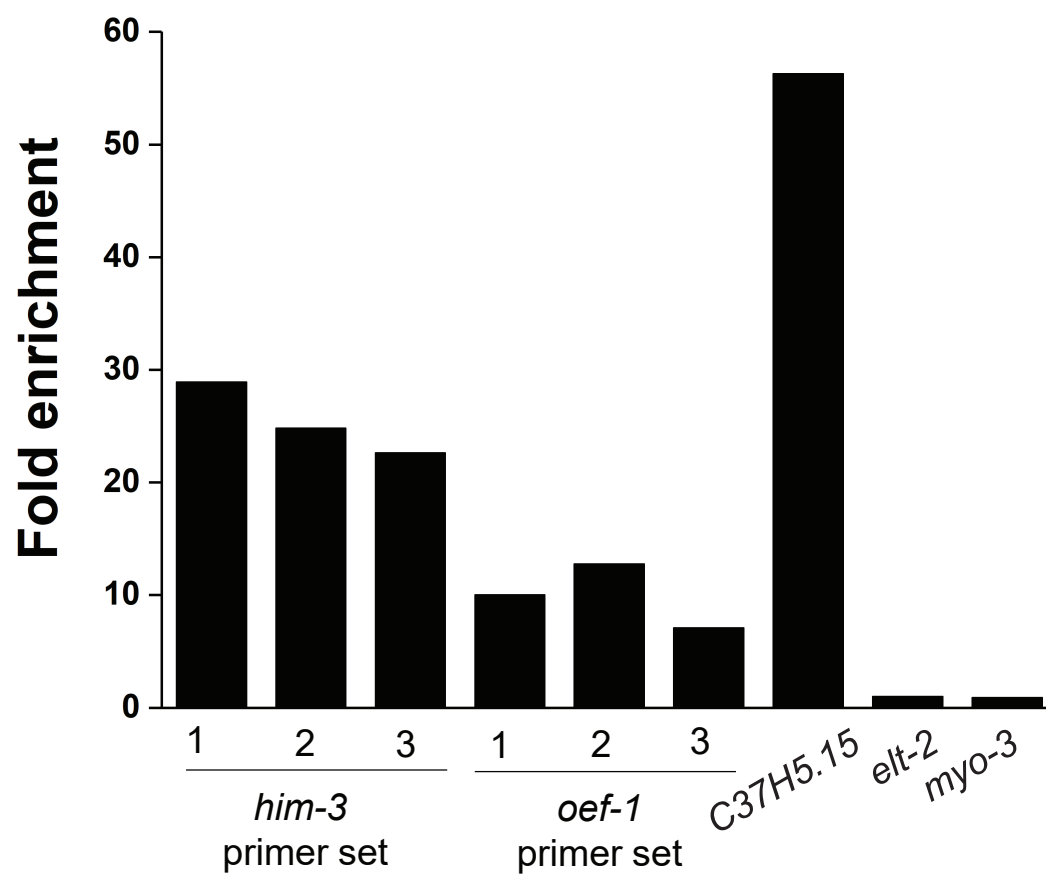**Figure S7**

Supplement: Supplementary file 7 — Figure S7 Confirmation of germline-enriched H3K27ac modification in isolated germ nuclei by ChIP-qPCR. Two previously characterized germline-expressed (him-3 and oef-1) and soma-expressed genes (elt-2 and myo-3) were examined for abundance of H3K27ac in IGN by ChIP-qPCR. Three sets of primers were tested for each germline-specific gene. C37H5.15 served as positive control. Elt-2 served as negative control and was used to calculate fold enrichment. ChIP results are expressed as percent of input using Ct values (A) and fold enrichment of H3K27ac modification normalized to elt-2 (B). (PDF 1136 kb) [file 12864_2019_5893_MOESM7_ESM.pdf]

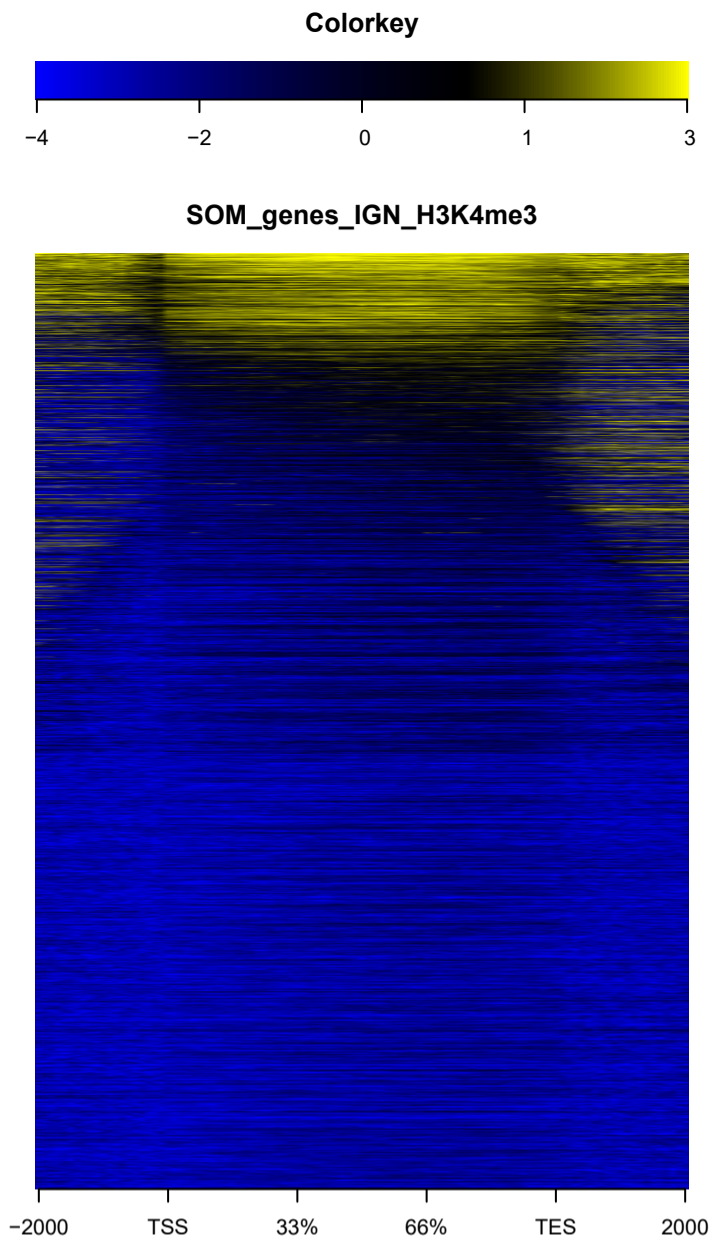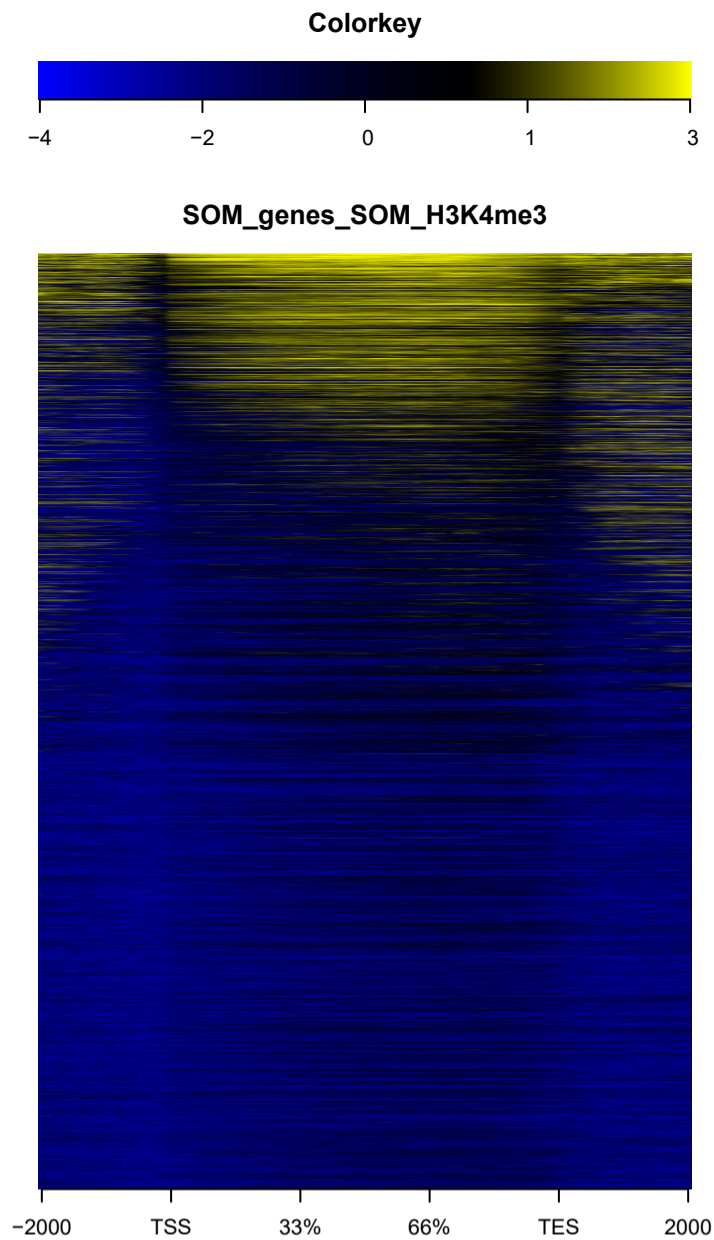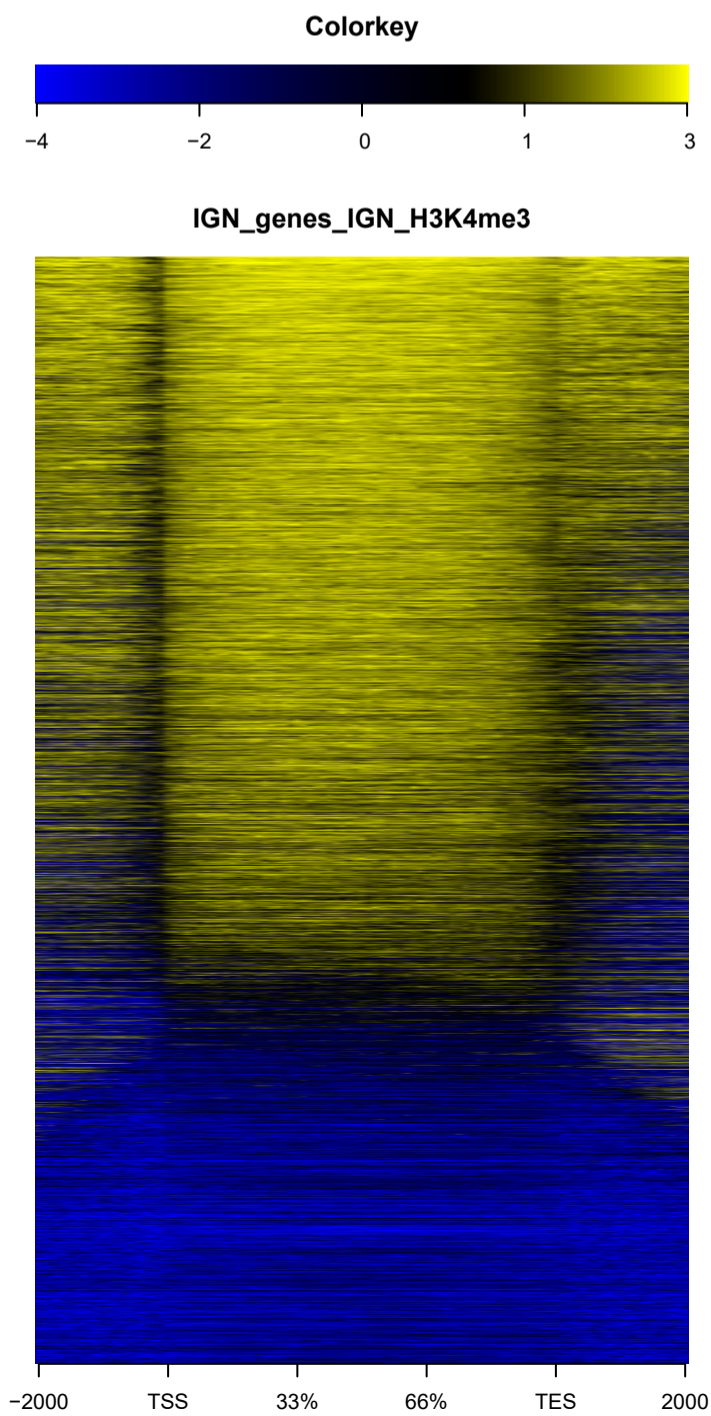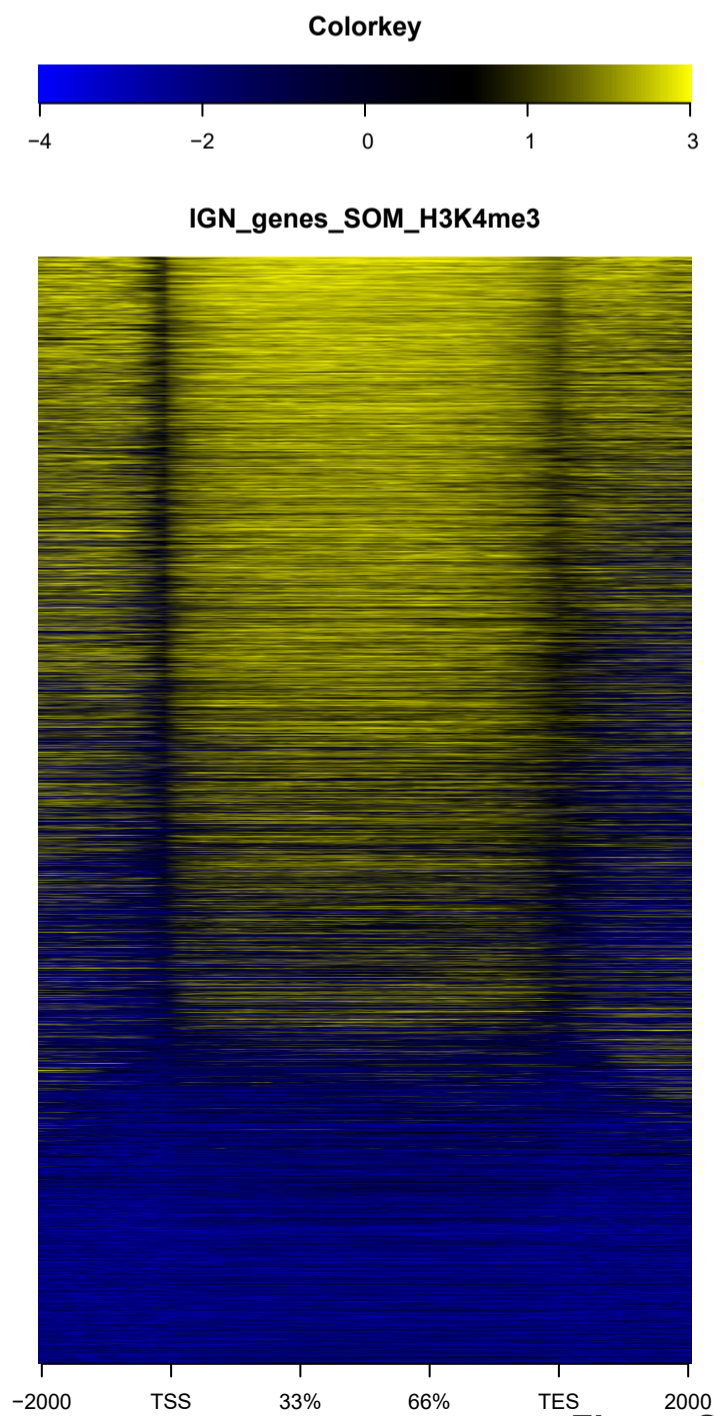

**Figure S8**

Supplement: Supplementary file 8 — Figure S8 Heatmap of H3K4me3 levels for genes with tissue-enriched expression. Heatmap displaying the levels of H3K4me3 for genes with SOM-enriched expression or IGN-enriched expression, as assayed by IGN H3K4me3 ChIP-seq and SOM H3K4me3 ChIP-seq. (PDF 3310 kb) [file 12864_2019_5893_MOESM8_ESM.pdf]
