# Supplementary material for: Isolated C. elegans germ nuclei exhibit distinct genomic profiles of histone modification and gene expression
Source: BMC Genomics. 2019 Jun 17;20:500. doi: 10.1186/s12864-019-5893-9 (PMC6580472; doi:10.1186/s12864-019-5893-9)
Supplement: Supplementary file 9 — Table S1 Quantification of fraction of germline nuclei (DOCX 14 kb) [file 12864_2019_5893_MOESM9_ESM.docx]

**Table S1 Quantification of fraction of germline nuclei**

GFP+; DAPI+ nuclei represent germline nuclei and GFP-; DAPI+ nuclei represent non-germline nuclei. Two independent experiments for each genotype were performed.

| **Strain** | **GFP +; DAPI +** | **GFP -; DAPI +** | **Total nuclei** |
| --- | --- | --- | --- |
| OEF-1::GFP | 1936 (91.02%) | 191 (8.98%) | 2127 (100%) |
| *pie-1*p::GFP::H2B | 1881 (89.1%) | 230 (10.9%) | 2111 (100%) |
